# Early Immunological Biomarkers for Personalized Treatment Selection in Severe COVID-19: Post Hoc Machine Learning Analysis of a Randomized Clinical Trial

**DOI:** 10.2196/78471

**Published:** 2026-06-04

**Authors:** Symeon Savvopoulos, Anastasia Papadopoulou, Georgios Karavalakis, Ioanna Sakellari, Grigorios Georgolopoulos, Christos Argyropoulos, Evangelia Yannaki, Haralampos Hatzikirou

**Affiliations:** 1 Department of Mathematics, Khalifa University of Science and Technology, Abu Dhabi, United Arab Emirates; 2 Faculty of Chemical Engineering and Technology, Cracow University of Technology, Cracow, Lesser Poland, Poland; 3 Hematology Department-Hematopoietic Cell Transplantation Unit, Gene and Cell Therapy Center, George Papanikolaou Hospital, Thessaloniki, Greece; 4 Department of Genetics, Development and Molecular Biology, School of Biology, Aristotle University of Thessaloniki, Thessaloniki, Greece; 5 Department of Chemical Engineering, King Fahd University of Petroleum and Minerals, Dhahran, Saudi Arabia; 6 Interdisciplinary Research Center for Refining & Advanced Chemicals, King Fahd University of Petroleum and Minerals, Dhahran, Saudi Arabia; 7 Center for Integrative Petroleum Research, King Fahd University of Petroleum and Minerals, Dhahran, Saudi Arabia; 8 Department of Medicine, University of Washington, Seattle, WA, United States; 9 Center for Information Services and High Performance Computing, Technische Universität Dresden, Willers-Bau building, at Zellescher Weg 12, Dresden, 01069, Germany, 49 351463-31943

**Keywords:** SARS-CoV-2-specific T cells, COVID-19 treatment, machine learning, risk stratification, severe COVID-19

## Abstract

**Background:**

Severe COVID-19 is a global health concern despite continuous vaccination campaigns because current therapies, such as dexamethasone and remdesivir, do not considerably improve immune function, especially in high-risk individuals. SARS-CoV-2–specific T cells (CoV-2-STs) from vaccinated or convalescent donors are a promising new treatment that can enhance clinical outcomes and viral-specific immunity. CoV-2-STs improve T cell proliferation and recovery without raising safety concerns, according to randomized studies. Targeting patients for immunotherapy is made more difficult by the variability in COVID-19 progression brought on by variables like age and comorbidities. In order to further enable precision medicine and patient care, machine learning techniques are being used to analyze clinical data, predict disease severity, and optimize treatment. However, their use in guiding the treatment of novel therapies like CoV-2-STs using early cellular immunology data is limited and requires improvement.

**Objective:**

The purpose of this research was to stratify high-risk individuals using early immunological and clinical indicators, and to develop a prediction tool to enable individualized treatment decisions, either with standard of care (SoC) or with a combination of CoV-2-STs and SoC.

**Methods:**

A randomized phase 1‐2 trial enrolled 87 patients with severe COVID-19 (CoV-2-STs+SoC: n=57; SoC only: n=30). We performed a post hoc machine learning analysis. Clinical and biomarker data from days 0 and 5 were analyzed longitudinally. Shrinkage linear discriminant analysis was used to create arm-specific prognostic models, and stratified cross-validation assessed performance (area under the receiver operating characteristic curve, area under the precision-recall curve, sensitivity, specificity, *F*_1_-score, and Brier score). To investigate arm-swap scenarios, Monte Carlo simulations (4 variance models) generated hypothetical early-treatment trajectories.

**Results:**

Day 5 analysis revealed a significant difference in CD3+, CD8+, CD56+, and CoV-2-STs between the 2 treatment groups (CoV-2-STs+SoC vs SoC). At Day 60, 64.9% (37/57) of patients with CoV-2-STs+SoC survived, compared with 40% (12/30) of patients with only SoC, resulting in a crude odds ratio of 2.8 (95% CI 1.1‐6.9) for recovery. SoC-only models had an area under the curve between 0.72 and 0.76, while CoV-2-STs+SoC models had an area under the curve between 0.86 and 0.88. Area under the precision-recall curve values for CoV-2-STs+SOC models were 0.74‐0.78, with sensitivity ranging from 0.89 to 0.91 and specificity from 0.83 to 0.87, compared with SoC-only models with a sensitivity of approximately 0.95 and a specificity of 0.58‐0.62. Simulation studies indicate that CoV-2-STs+SoC may benefit up to 30% (approximately 9/30) of patients receiving SoC alone. Misclassifying candidates with CoV-2-STs+SoC as SoC-only could increase critical outcomes by up to approximately 22%.

**Conclusions:**

A robust computational tool for severe COVID-19 risk stratification and treatment selection is presented. Precision medicine and early treatment outcome prediction are supported by clinical and immunological data integration. Prospective studies are needed to confirm its clinical utility.

## Introduction

SARS-CoV-2–specific T cell (CoV-2-ST) responses can be elicited by vaccination, natural infection, or their combination (hybrid immunity) [1-3] and have been proven crucial in controlling and eliminating the virus [4,5]. Previous research, including our own, has shown the feasibility of ex vivo expansion of virus-specific T cells from the long-lived T cell memory compartment of donors who have previously developed immunity against various viruses (adenovirus, cytomegalovirus, Epstein-Barr virus, BK virus, and human herpesvirus-6), primarily in the context of hematopoietic cell or solid organ transplantation. Likewise, and outside the transplantation setting, CoV-2-STs have been expanded from convalescent or vaccinated donors [1,5-11].

The safety and efficacy of the adoptive transfer of CoV-2-STs was recently demonstrated by our group in a 2:1 randomized phase 1 and 2 clinical trial implemented during the delta variant (B.1.617.2) predominance [12]. In this study, patients with severe COVID-19 were enrolled, who either received CoV-2-STs along with the standard-of-care (SoC), including dexamethasone and remdesivir, or only the SoC. The patients treated with CoV-2-ST+SoC showed higher rates of and faster recovery from severe COVID-19, improved overall survival, and significant in vivo expansion of CoV-2-STs as compared with patients treated with SoC.

Machine learning (ML) algorithms enable the mapping of multiple inputs to a desired output [13,14]. Their ability to identify nonlinear relationships allows ML models to have an improved prediction. In recent years, the integration of various ML models in the analysis of feature importance and classification of multidimensional heterogeneous datasets has provided promising tools toward trend prediction and risk stratification of patients with COVID-19, as well as optimization of current medical practices [15-17]. ML applications have been explored for identifying epidemics, differentiating COVID-19 pneumonia from other types, estimating severity risk, triaging patients, and forecasting prognoses [18]. In particular, investigators have used artificial intelligence (AI), and in particular deep learning, to early detect and differentiate COVID-19 from other pneumonias via chest computed tomography scans with increased sensitivity and accuracy, often surpassing nucleic acid tests and improving radiologists’ performance metrics [19,20]. Clinical AI is increasingly embedded in decision support to improve diagnosis, risk stratification, and treatment planning, but real-world uptake hinges on transparent, usable explanations and rigorous evaluation [21]. A broad synthesis across clinical domains shows model-agnostic explanation tools are common while gaps remain in explanation fidelity, clinician trust, and usability [21]. Complementing this, a capability or architecture view situates today’s systems as predominantly narrow, limited-memory tools trained on historical data, with theory-of-mind or general AI concepts still aspirational and raising governance and regulatory questions [22]. Together, these perspectives motivate interpretable methods, standardized metrics, and clinically grounded validation for safe deployment.

Using ML, other groups have developed risk models to predict COVID-19 mortality risk or triage patients upon hospital admission [23]. Collectively, these studies underscore the potential of computational tools to augment traditional medical approaches (ie, imaging) and data analysis methods toward improved diagnosis, risk assessment, and patient management. Despite introducing bias and poor reporting in the current literature of numerous COVID-19 prediction models, as critically reviewed by Wynants and colleagues [24], they identified a handful of models with good predictive performance and at low risk of bias at external validation.

Our aim, herein, is to use a post hoc ML analysis to identify critical biomarkers associated with specific COVID-19 treatment outcomes and to predict severely affected individuals unlikely to benefit from the SοC-only treatments, including dexamethasone and remdesivir. Based on these biomarkers, we subsequently developed a novel computational tool enabling the prediction of recovery from severe COVID-19 after the administration of either SοC-only or adoptive immunotherapy with CoV-2-STs in patients with high risk, within the first days of their admission to the hospital. This tool allows patient risk stratification and identification of the most suitable candidates for cellular immunotherapy, facilitating precise decision-making.

## Methods

### Dataset

No clinical trials have been conducted for this study. The data used in this study were obtained from the CoV-2-STs-001 study, assessing the safety and efficacy of CoV-2-STs compared with the SoC in hospitalized adults with severe COVID-19 during delta variant predominance [12]. In this trial, hospitalized patients with high-risk of COVID-19, within the first 6 days of symptom onset, with pneumonia or oxygen saturation on air, equal to or less than 94%, CD3+ cells less than 650 cells/μL, and at least 1 elevated serum biomarker (C-reactive protein [CRP], lactate dehydrogenase [LDH], ferritin, and D-Dimers) were randomly assigned (2:1) to receive either CoV-2-STs and SoC (CoV-2-STs-group; arm A: n=57 patients) or SoC-alone (SoC-group; arm B: n=30) and were followed for 60 days. Both treatment arms received the same SoC. The same baseline SoC, which comprised dexamethasone and remdesivir as part of the recommended therapy for hospitalized patients with severe COVID-19, was given to both the CoV-2-ST+SoC and SoC-only groups. Comparability was maintained as the investigation found no appreciable variations in how these therapies were administered between the 2 groups. However, depending on clinical assessment, there were some differences in the usage of extra supportive therapies. At the clinician’s discretion, several patients in the SoC group received tocilizumab when their cytokine levels continued to be high.

The cells were infused within a maximum of 24 h after randomization (day 0 for both arms). Clinical data were assessed and recorded daily. Laboratory assessments, including (1) levels of CRP, LDH, ferritin, D-Dimers, and white blood cells (WBCs); (2) number of circulating CD3+, CD4+, CD8+, and CD56+ cells; and (3) CoV-2-STs, SARS-CoV-2–specific neutralizing antibodies (SARS-CοV-2-NAbs), and serum cytokine measurements, were performed on days 0, 3, 5 (±2), 10 (±2), 15 (+3), 22 (+3), 30 (+3), 45 (+3), and 60 (+7).

Serum cytokines (interleukin [IL] 6) and tumor necrosis factor-α (TNF-α) were tested to monitor systemic inflammation in patients. The frequency of CoV-2-STs was measured using interferon-gamma (IFN-γ) enzyme-linked immunospot (ELISpot) assays, which detect IFN-γ production after antigen stimulation. Direct ex vivo ELISpot testing showed T cells’ functional responsiveness to SARS-CoV-2 antigens.

For the scope of this study, only the biomarker values assessed on days 0 and 5, and their correlation with the outcomes of recovery (defined as a patient score ≤3 in the 8-category World Health Organization [WHO] ordinal scale) and survival were considered [25]. The patient population was subjected to an “as treated” analysis, including in arm B, 1 patient who was initially randomized to arm A but was intubated before receiving the CοV-2-STs (arm A, CoV-2-STs+SoC n=57 patients, and arm B, SoC-group n=30 patients).

### Computational Methodology

The primary objective of this research endeavor was to develop, by using robust data analysis techniques, a medical tool capable of predicting with high accuracy the ultimate treatment outcomes of severe COVID-19. The treatment modalities assessed were the SοC alone (including dexamethasone and remdesivir) or the adoptive transfer of CοV-2-STS in addition to SοC, from the randomization of patients (day 0) until study completion (day 60).

**Figure 1.**
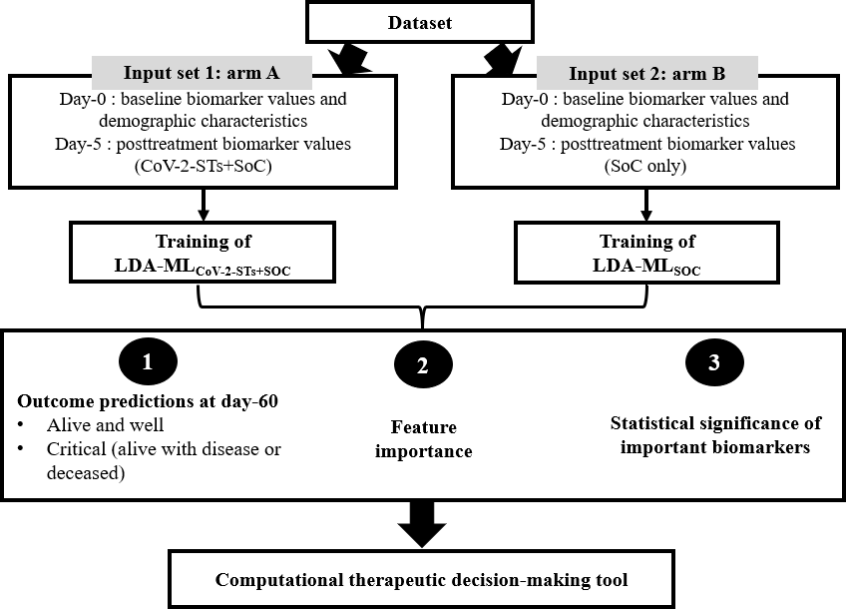
Workflow diagram. This diagram depicts the steps for predicting the treatment response and designing the computational, therapeutic decision-making tool. CoV-2-ST: SARS-CoV-2–specific T cell; LDA: linear discriminant analysis; ML: machine learning; SoC: standard-of-care.

Our strategy involved a multistep process outlined in Figure 1. Initially, we pinpointed a critical, early postenrollment time point (day 5) where significant biomarker differences were observed between the 2 study groups (arms A and B), thus implying that the treatment impact was visible by day 5 on patients’ biomarker profiles. These differences were detected by analyzing the biomarkers’ descriptive statistics at baseline and on day 5 and calculating the rate of change for each biomarker over the initial 5 days. We evaluated a panel of supervised learners—L2-regularized logistic regression, penalized or shrinkage linear discriminant analysis (LDA), random forest, and gradient boosting—to predict day-60 outcome from day-5 measurements. The end point was defined a priori as a binary composite: favorable (alive in remission) versus critical (deceased or alive-with-disease). Modeling was performed separately by treatment arm. All preprocessing occurred within cross-validation (CV) folds to prevent leakage. Numeric features were median-imputed and standardized (*z* scores); categorical features were mode-imputed and one-hot encoded. We used the same stratified CV folds for all models with nested hyperparameter tuning [26,27].

To substantiate model choice, we compared 5 different models, including LDA solved via singular value decomposition (svd), shrinkage or regularized LDA, regularized logistic regression, random forest, and gradient boosting using identical stratified k-fold CV splits for all methods [28,29]. Within each training fold, we performed inner-fold tuning, and all preprocessing (imputation and standardization) was confined to the training data to prevent leakage. We reported out-of-fold (OOF) performance for each model, including area under the receiver operating characteristic curve (AUROC), area under the precision-recall curve (AUPRC), Brier score, sensitivity, specificity, and *F*_1_-score.

Feature attribution was computed within CV folds and then aggregated across folds. For logistic regression, the standardized coefficients could be used. For shrinkage-LDA, the standardized discriminant loadings from linear discriminant 1 (and linear discriminant 2, where defined) could be reported as the primary importance measure. For tree-based models (random forest and gradient boosting), model-agnostic permutation importance and Shapley Additive Explanations values using fold-fitted models could be computed [30]. In our case, based on the best model selected, the cross-model concordance of top features is summarized, and univariate confirmation (Kruskal-Wallis [KW] for continuous variables; chi-square for categorical) is provided to contextualize multivariable findings [31,32]. Using the above workflow, we trained 2 arm-specific models: ML(CoV-2-STs+SoC) for arm A and ML(SoC-only) for arm B, each using day-5 predictors. Comparative performance summaries and calibration diagnostics are presented in the Results section.

The ultimate goal of our efforts was the development of a computational tool capable of predicting a patient’s most probable outcome based on their initial characteristics and the treatment applied, thus enabling optimal therapeutic decision-making. Using this tool, we conducted simulations to assess the potential benefits of administering CoV-2-ST treatment to patients who initially received only the SoC (arm B) and the potential adverse outcome of treating with SoC only to patients who had initially received CoV-2-STs. For these simulations, we applied a Monte Carlo method to estimate day-5 biomarker values, considering the distribution of biomarker actual rates of change from day 0 to day 5, in each group. This approach allowed us to generate 1000 hypothetical scenarios for each group and predict clinical outcomes at day 60 using our ML(SoC-only) and ML(CoV-2-STs+SoC) models, respectively. Our approach for both ML models predicting patients’ outcomes with CoV-2-STs+SoC or SoC-only treatment is summarized in Figure 1.

### ML Method

The input data for each group encompassed demographic, clinical, and laboratory parameters, including age, gender, Karnofsky score (KS), comorbidities, serum levels of IFN-γ, IL-10, IL-2, IL-6, TNF-α, SARS-CοV-2-NAbs, circulating CD3+, CD4+, CD56+, CD8+, and CoV-2-STs, and levels of CPK, CRP, D-Dimers, ferritin, LDH, and WBC. The dataset was subjected to standardization using the Standard Scaler. The correlations between the features were quantified (Figure S1 in Multimedia Appendix 1). However, highly correlated features were not removed because LDA classifications transform the input variables into a set of orthogonal uncorrelated variables [33]. Also, it is known that collinearity does not impact the performance of the LDA classification. Both SoC and CoV-2-STs groups, k-fold CV with k=5 was used, ensuring robust evaluation by partitioning the dataset into 5 subsets and iteratively using 4 folds for training and 1 for validation in each iteration. For the classification task, the classifier was used based on patients’ day-5 biomarker levels, along with baseline characteristics, such as age, comorbidities, and KS, and used to categorize the patients into distinct outcomes, such as (1) alive and well, (2) deceased, and (3) alive with disease. Among disease severity variables (KS, oxygen supply, and 8-point category WHO Ordinal Scale), KS was selected to be incorporated in the classification model, as all variables were highly correlated [12,34]. The model performance was assessed using the following metrics: AUPRC, AUROC, sensitivity, specificity, *F*_1_-score, and Brier score. Because the outcome is imbalanced, we prespecified AUPRC as the primary discrimination metric. We also report AUROC as a threshold-free complement. To summarize performance at an actionable operating point, we compute sensitivity, specificity, precision or recall, and *F*_1_-score at the fold-wise threshold that maximizes *F*_1_-score on the validation split. Calibration is assessed with the Brier score. We summarize metrics as mean (SD) across the same stratified CV folds. Accuracy is not used as a headline metric because it can be inflated by the majority class in imbalanced datasets.

### Statistical Analysis

For categorical variables, prevalence and percentage values were used to calculate descriptive statistics. Medians and IQR were used for continuous variables, and median and range were used for variables with skewed distributions. For nonparametric comparisons, the KW and Mann-Whitney *U* tests were used [31]. The Fisher exact test or the chi-square test, as appropriate, was used to test categorical comparisons [34].

### Ethical Considerations

The data used in this study were obtained from the CoV-2-STs-001 study (Clinical Trials.gov NCT05447013, EudraCT:2021-001022-22), which was conducted in accordance with the Declaration of Helsinki. Approval for the clinical trial was obtained from the National Organization for Medicines and jurisdictional ethics committees (the National Ethics Committee and the institutional review boards of George Papanikolaou Hospital and Hippokrateion Hospital; IRB 5th/03-06-2021) after thorough discussions and protocol adjustments on eligibility criteria and safety assessments and an interim and final review of the phase 1 study data (National Ethics Committee) before beginning the phase 2 study. Continuous safety surveillance by pharmacovigilance was also integrated into all study phases.

Written informed consent was obtained from all participants before enrollment, including consent for the use of deidentified clinical and biomarker data for subsequent research analyses. All data used in this post hoc ML analysis were fully anonymized before analysis, and no personally identifiable information was accessible to the investigators performing the computational analyses, thereby ensuring participant privacy and confidentiality. Participants did not receive financial compensation for participation in the present secondary analysis.

## Results

### Time Point Selection: Identification of the Key Time Point Impacting Clinical Outcome

**Figure 2.**
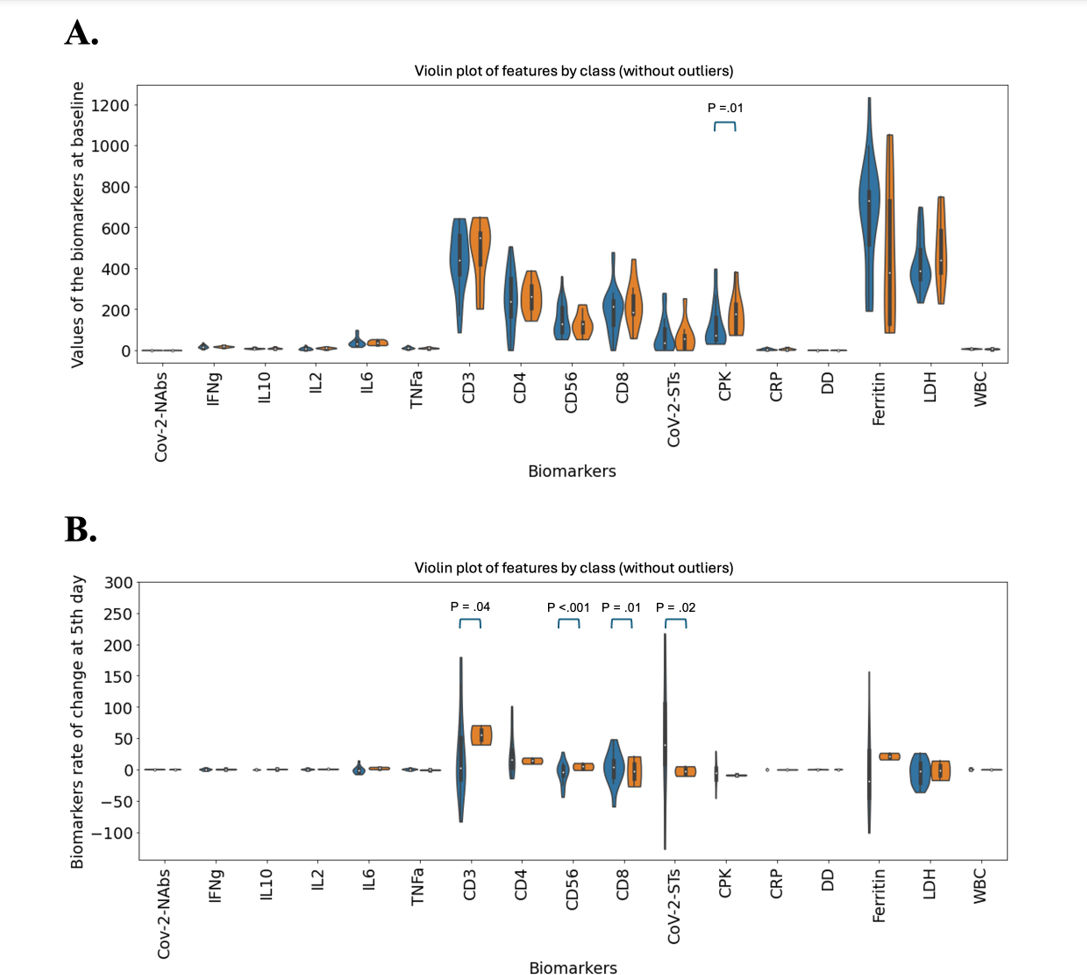
(A) Values of the biomarkers at baseline and (B) rate of change of biomarkers between day-5 and baseline. The *P* values of the most important features estimated with Mann-Whitney *U* test. CD: cluster of differentiation; CoV-2-ST: SARS-CoV-2–specific T cell; CPK: creatine phosphokinase; CRP: C-reactive protein; DD: D-dimer; IFNg: interferon-gamma; IL: interleukin; LDH: lactate dehydrogenase; NAb: neutralizing antibody; SoC: standard-of-care; TNFa: tumor necrosis factor-α; WBC: white blood cell.

The baseline demographic and clinical characteristics of study participants were well-balanced between the 2 groups and are summarized in Table 1. The corresponding intention-to-treat analysis has been reported in detail elsewhere [12]. Importantly, while at baseline there were no differences in the levels of tested biomarkers between patients treated with CoV-2-STs+SoC and SoC, apart from CPK (*P*=.01; Figure 2A), on day 5 post infusion, the rate of changes in the levels of CD3+ T cells, natural killer cells (CD56+ natural killer [NK] cells), CD8+ T cells, and CoV-2-STs and from baseline, were significantly different between the 2 arms (Figure 2B), with increases favoring the CoV-2-ST group (Mann-Whitney *P* values of .04, <.001, .01, and .02, respectively).

**Table 1.** Baseline characteristics of study participants.

Characteristics	Arm A (n=57), n (%)	Arm B (n=30), n (%)	*P* value
Age (y; >50)	42 (73.7)	25 (83.3)	.30
Sex			.97
Female	23 (40.4)	12 (40)	
Male	34 (59.6)	18 (60)	
Oxygen supply			.78
No	2 (3.5)	0 (0)	
Yes	55 (96.5)	30 (100)	
Oxygen supply specification			.35
Nasal cannula	18 (33.3)	8 (26.7)	
Venturi mask 35%	1 (1.8)	0 (0)	
Venturi mask 40%	2 (3.5)	2 (6.7)	
Venturi mask 60%	10 (17.5)	2 (6.7)	
Nonrebreather mask	15 (26.3)	8 (26.7)	
Nonrebreather mask+nasal cannula	1 (1.8)	3 (10)	
High flow	5 (8.8)	3 (10)	
Noninvasive mechanical ventilation	2 (3.5)	4 (13.3)	
Intubation-mechanical ventilation	1 (2)	0 (0)	
Karnofsky score			.45
90‐100	10 (17.5)	3 (10)	
70‐80	29 (50.9)	14 (46.7)	
50‐60	18 (31.6)	13 (43.3)	
Comorbidities			.13
None	12 (21.1)	4 (13.3)	
1	18 (31.6)	7 (23.3)	
2	15 (26.3)	6 (20)	
≥3	12 (21)	13 (43.3)	
Vaccination			.26
No	47 (82.5)	22 (73.3)	
Yes	10 (17.5)	8 (26.7)	
Patient outcome			—^a^
Alive and well	37 (64.9)	12 (40)	
Critical	20 (35.1)	18 (60)	

a Not applicable.

The distinctive immunological responses elicited by CoV-2-STs as opposed to SoC treatment on day 5 identified this day as a critical time point. On day 5, CoV-2-STs selectively modulated certain biological markers, leading to enhanced adaptive immunity (as evidenced by the increases in T cells and CoV-2-STs) while preserving innate immunity (indicated by maintained levels of NK cells). The day-5 data subsequently played an instrumental role in the development of our ML model.

To gain deeper insights into how the 2 treatments may differentially modulate the immune response and the biomarker components interact, we analyzed the correlations among all biomarkers on day 5 post treatment for each group (Figure S1 in Multimedia Appendix 1). As expected, a significant correlation (exceeding 0.7) between CD3+ and CD4+ cells was observed in both groups, suggesting a predominant presence of helper T cells in circulation. This aligns with the established critical role of CD4+ T cells in immune regulation and viral clearance in COVID-19. In group A, the observed correlation between IFN-γ and IL-2 indicates a robust T cell response. IL-2 is essential for T cell proliferation, while IFN-γ is a crucial cytokine produced by activated T cells. This correlation implies that the administration of CoV-2-STs enhances T cell–mediated immune defense against the virus. Conversely, in group B, significant correlations of proinflammatory cytokines IFN-γ and IL-6 suggest an innate and broad inflammatory reaction aiming at combating the virus and resolving acute inflammation. Additionally, the correlations between proinflammatory and anti-inflammatory cytokines, such as IFN-γ and IL-10 or IL-6 and IL-10, may represent mechanisms to mitigate potential collateral damage from an intense immune response. Furthermore, a strong correlation between CPK and TNF-α was observed, linking muscle impairment in severe COVID-19, such as myalgia, rhabdomyolysis, or myositis, to a stress response involving proinflammatory TNF-α [35-37]. Overall, these findings underscore qualitatively distinct immune modulations by the CoV-2-ST treatment compared with SoC.

### Patient Classification and Feature Importance With Respect to Clinical Outcomes

Our objective was to use early posttreatment biomarkers from day 5 to predict day 60 clinical outcome and highlight markers most informative for prognosis. Preprocessing included fold-wise transformation and standardization to avoid leakage. Because of severe class imbalance and to stabilize estimation, we defined a binary composite end point with favorable (alive and healthy) versus critical (deceased or alive-with-disease). Models were fit with stratified CV and evaluated separately within each cohort or treatment arm.

We first benchmarked regularized logistic regression, shrinkage-LDA, random forest, and gradient boosting using the same stratified CV folds with nested tuning. We report AUROC, AUPRC (primary), sensitivity, specificity, *F*_1_-score, and Brier, with CIs from repeated CV and calibration curves. The index model was chosen as the classifier with the highest mean AUPRC and competitive calibration (Brier). Feature importance was then quantified on the index model using the method appropriate to its class—standardized coefficients for regularized logistic regression; signed, standardized discriminant loadings for shrinkage-LDA; and Shapley Additive Explanations with in-fold permutation importance for tree-based models—with cross-model concordance. On arm A (Table 2), shrinkage-LDA performs competitively with the best nonlinear alternative. Its AUROC (mean 0.874, SD 0.052) is within fold-to-fold variability of random forest (mean 0.881, SD 0.079), with AUPRC and *F*_1_-score similarly close (0.832 and 0.842, respectively), and a respectable Brier score (0.137). Thus, shrinkage-LDA is not dominated by the more complex models while retaining a transparent linear decision rule. On arm B (Table 3), random forest attains the highest mean discrimination and *F*_1_-score (mean AUROC 0.739, SD 0.042; mean AUPRC 0.862, SD 0.077; mean *F*_1_-score 0.836, SD 0.048), whereas shrinkage-LDA delivers the highest sensitivity (mean 0.950, SD 0.112) at the cost of lower specificity and a higher Brier (mean 0.294, SD 0.101). Given the small-n setting and class imbalance, this sensitivity-favoring profile may be desirable when false negatives are clinically costly, and we therefore report shrinkage-LDA alongside the index model. Because false negatives are costly, the best model was specified as the one that achieves noninferior discrimination and calibration (within CV variability) and the highest sensitivity at a clinically relevant threshold. Shrinkage-LDA satisfies this on arm A and yields the highest sensitivity on arm B, coupled with its interpretability and regularization. Shrinkage-LDA was selected as the primary clinical model and reports its signed, standardized loadings as the main feature-importance summary. KS was chosen as a representative variable over other disease severity variables, as mentioned earlier. While random forest offered superior overall discrimination (AUROC 0.739), we prioritized shrinkage-LDA for the clinical decision tool because, in the context of severe COVID-19, the “cost” of a false negative (missing a patient who will die) is far higher than a false positive (overtreating a patient who would have recovered). LDA’s near-perfect sensitivity (0.95) ensures patients with high risk are captured, even at the expense of lower specificity. Factors were initially ranked in terms of importance for each group, followed by further filtering to consider only those that significantly differed between the 2 outcome groups (alive and well, and critical) by KW test (Table S1 in Multimedia Appendix 1). According to this analysis, key determinants for the day-60 outcome in the CoV-2-STs+SoC arm included (1) the KS; (2) the levels of CD3+, CD4+, CD8+, and CD56+ cells; (3) the levels of circulating CoV-2-STs; and (4) the levels of IL-6, LDH, ferritin, CRP, and WBC, while in the SoC-arm only the circulating CoV-2-STs, CD3+, CD56, and the CRP levels were identified as significant (Table S1, Figures S2 and S3 in Multimedia Appendix 1). Based on these data, we proceeded to further analyses to define suggested cutoffs for critical variables, presented in Table 4, according to the corresponding treatment and outcome.

**Table 2.** Model comparisons for CoV-2-STs^a^+SoC^b^ arm A (mean [SD] across folds).

Model	AUROC^c^, mean (SD)	AUPRC^d^, mean (SD)	Sensitivity, mean (SD)	Specificity, mean (SD)	*F*_1_-score, mean (SD)	Brier, mean (SD)
Random forest	0.881 (0.079)	0.858 (0.116)	0.850 (0.137)	0.975 (0.056)	0.892 (0.062)	0.126 (0.029)
LDA^e^ (shrinkage)	0.874 (0.052)	0.832 (0.119)	0.800 (0.112)	0.946 (0.074)	0.842 (0.053)	0.137 (0.054)
Gradient boosting	0.835 (0.162)	0.753 (0.234)	0.850 (0.224)	0.893 (0.147)	0.827 (0.167)	0.135 (0.127)
LogReg^f^ (L2)	0.755 (0.045)	0.696 (0.111)	0.850 (0.137)	0.754 (0.175)	0.746 (0.070)	0.193 (0.060)
LDA (svd^g^)	0.737 (0.040)	0.676 (0.101)	0.850 (0.137)	0.757 (0.146)	0.741 (0.012)	0.213 (0.036)

a CoV-2-ST: SARS-CoV-2–specific T cell.

b SoC: standard-of-care.

c AUROC: area under the receiver operating characteristic curve.

d AUPRC: area under the precision-recall curve.

e LDA: linear discriminant analysis.

f LogReg: logistic regression

g svd: singular value decomposition.

**Table 3.** Model comparisons for SoC^a^ arm (mean [SD] across folds).

Model	AUROC^b^, mean (SD)	AUPRC^c^, mean (SD)	Sensitivity, mean (SD)	Specificity, mean (SD)	*F*_1_-score, mean (SD)	Brier, mean (SD)
Random forest	0.739 (0.042)	0.862 (0.077)	0.850 (0.137)	0.800 (0.298)	0.836 (0.048)	0.251 (0.062)
LDA^d^ (shrinkage)	0.667 (0.200)	0.821 (0.110)	0.950 (0.112)	0.500 (0.373)	0.831 (0.055)	0.294 (0.101)
Gradient boosting	0.642 (0.120)	0.822 (0.086)	0.883 (0.162)	0.467 (0.506)	0.801 (0.038)	0.215 (0.032)
LogReg^e^ (L2)	0.458 (0.267)	0.605 (0.093)	1.000 (0.000)	0.100 (0.224)	0.764 (0.096)	0.497 (0.157)
LDA (svd^f^)	0.354 (0.340)	0.641 (0.190)	0.950 (0.112)	0.300 (0.447)	0.776 (0.105)	0.534 (0.273)

a SoC: standard-of-care.

b AUROC: area under the receiver operating characteristic curve.

c AUPRC: area under the precision-recall curve.

d LDA: linear discriminant analysis.

e LogReg: logistic regression

f svd: singular value decomposition.

**Table 4.** Suggested cutoffs of the most important key features between alive and deceased patients.

Group and biomarker	Most probable threshold values in alive patients	Most probable threshold values in critical patients
CoV-2-STs^a^+SoC^b^-arm		
IL-6^c^ (pg/mL)	≤100	>100
CD3^d^ (cells/μL)	≥500	<500
CD4 (cells/μL)	≥400	<400
CD56 (cells/μL)	≥100	<100
CoV-2-STs (SFC^e^/5×10^5^ PBMCs^f^)	≥200	<200
CRP^g^ (g/dL)	≤10	>10
LDH^h^ (units/L)	≤410	>410
Karnofsky	<3	≥3
CD8 (cells/μL)	≥150	<150
WBC^i^ (×10^3^ cells/μL)	≤12	>12
SoC-arm		
CD3 (cells/μL)	≥270	<270
CoV-2-STs (SFC/5×10^5^ PBMCs)	≥136	<136
CRP (g/dL)	≤5	>5
CD56 (cells/μL)	≥90	<90

a CoV-2-ST: SARS-CoV-2–specific T cell.

b SoC: standard-of-care.

c IL-6: interleukin 6.

d CD: cluster of differentiation

e SFC: spot forming cells.

f PBMC: peripheral blood mononuclear cell.

g CRP: C-reactive protein.

h LDH: lactate dehydrogenase.

i WBC: white blood cell.

### Binary End Point, Validation, and Imbalance Handling in Both Arms

Given the limited sample size and class imbalance, we modeled a binary end point—favorable (alive and well) versus critical (deceased or alive with disease)—using repeated stratified 5-fold CV (10 repeats) with identical splits across strategies and all preprocessing confined to the training fold. We evaluated shrinkage-LDA under 3 data-retaining imbalance strategies: empirical priors (no resampling), balanced priors (priors=0.5-0.5; LDA analog of class weighting), and Synthetic Minority Oversampling Technique (SMOTE) after one-hot (pragmatic oversampling). In each fold, the *F*_1_-optimal threshold was chosen on the validation split; training or validation confusion matrices were computed and aggregated. Because of imbalance, AUPRC was prespecified as the primary metric; we also report AUROC, sensitivity, specificity, precision, *F*_1_-score, and Brier, with 95% bootstrap CIs (B=2000) computed on OOF pairs while refitting the threshold within each bootstrap. Arm-specific OOF summaries, confusion matrices, and fold means are in Tables S2-S4 in Multimedia Appendix 1 (arm A) and Tables S5-S7 in Multimedia Appendix 1 (arm B).

In the CoV-2-STs+SoC arm (n=57), OOF discrimination was similar across modeling strategies (Table S2 in Multimedia Appendix 1): Brier=0.14‐0.16, AUROC=0.860‐0.880 (overlapping 95% CIs), and AUPRC=0.740‐0.780. At the *F*_1_-optimized operating point (Table S3 in Multimedia Appendix 1), combined validation rates were strong and well-balanced (*F*_1_-score=0.810‐0.840 with precision=0.740‐0.790, specificity=0.830‐0.870, and sensitivity=0.890‐0.910). Fold-level summaries (means and SD; Table S4 in Multimedia Appendix 1) recapitulated these patterns. Among priors, balanced priors tended to yield the highest sensitivity (approximately 0.910), whereas empirical priors offered slightly higher precision and *F*_1_-scores (approximately 0.83‐0.84). SMOTE after One-Hot Encoding performed comparably to these settings and did not provide a clear advantage.

In the SoC arm (n=30), OOF performance again showed overlapping CIs across strategies (Table S5 in Multimedia Appendix 1), with AUPRC=0.78‐0.81, AUROC=0.72‐0.76, and Brier=0.22‐0.27. Aggregated validation confusion (Table S6 in Multimedia Appendix 1) emphasized very high sensitivity (approximately 0.95 across strategies) at the cost of lower specificity (0.58‐0.62), resulting in precision=0.77‐0.79 and *F*_1_-score=0.85‐0.87. Fold-level means (Table S7 in Multimedia Appendix 1) were consistent (AUPRC=0.86‐0.88, AUROC=0.72‐0.75, Brier=0.25‐0.27, and *F*_1_-score=0.86‐0.87). Balanced and empirical priors were nearly indistinguishable, and SMOTE after One-Hot Encoding again produced no material gain—an overall profile that is expected given the smaller, more imbalanced sample.

**Figure 3.**
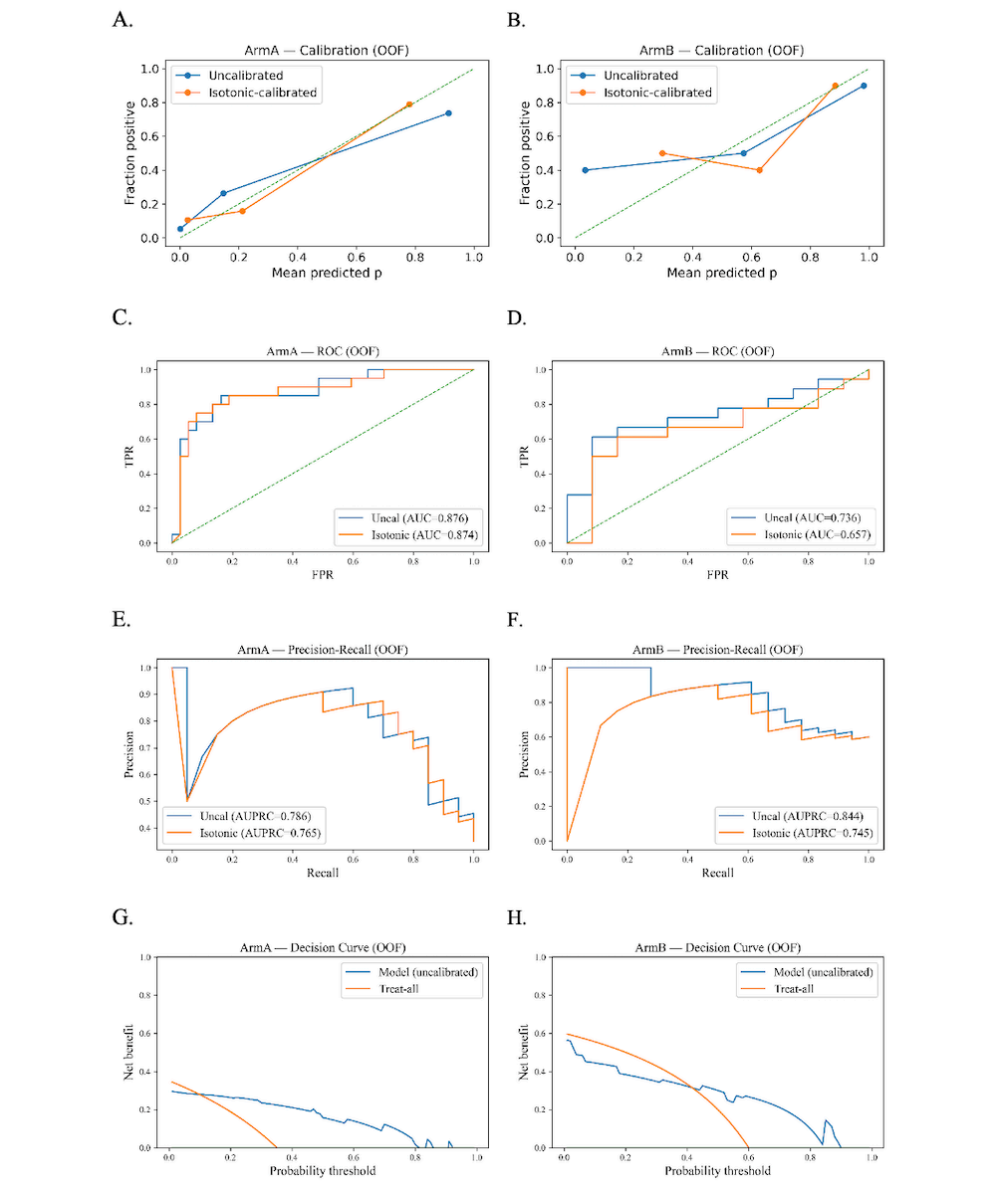
Out-of-fold performance visualizations. (A) and (B) Calibration (uncalibrated vs isotonic), (C) and (D) receiver operating characteristic curves with chance line, (E) and (F) precision-recall curves, and (G) and (H) decision curves comparing the model with “treat-all” and “treat-none.” Left panels correspond to SARS-CoV-2–specific T cell+standard-of-care (arm A) and right panels to standard-of-care (arm B). AUC: area under the curve; AUPRC: area under the precision-recall curve; OOF: out-of-fold; ROC: receiver operating characteristic curve; TPR: true positive rate; FPR: false positive rate.

These quantitative results align with the OOF visualizations in Figure 3. Calibration plots (A and B) show predicted probabilities tracking observed risk, while receiver operating characteristic (C and D) and precision-recall curves (E and F) indicate good separation in arm A and fair-to-good separation in arm B. Decision-curve analyses (G and H) demonstrate positive net benefit for model-guided decisions over “treat-none” and over “treat-all” across clinically relevant threshold ranges, supporting the practical value of risk-based treatment selection in both arms.

### Computational Therapeutic Decision-Making: A Robust Tool for Outcome Prediction

**Figure 4.**
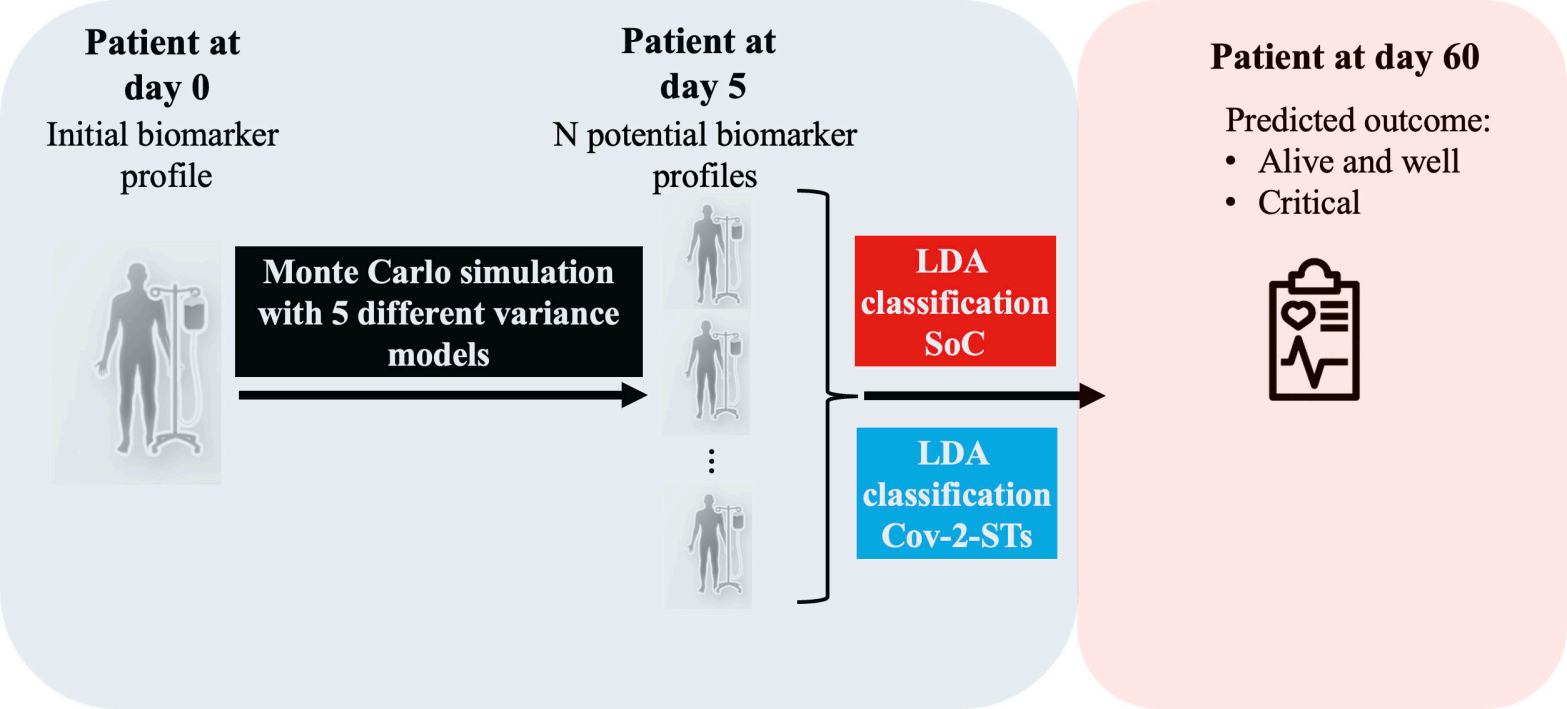
Clinical decision-making support tool design and workflow. CoV-2-ST: SARS-CoV-2–specific T cell; LDA: linear discriminant analysis; SoC: standard-of-care.

After fitting the 2 arm-specific models, LDA-ML_CoV-2-STs+SoC_ and LDA-ML_SoC_, we derived a predictive “what-if” tool to estimate how each patient’s predicted day-60 outcome might change under the alternative treatment (Figure 4). This analysis is associational and does not identify causal effects. Also, this tool will enable the identification of patients who have low probabilities to recover with SoC-only, while representing the best candidates for adoptive immunotherapy with CoV-2-STs or other alternative treatments. As an input, we used the baseline characteristics and biomarker values of 21 predictors in total (age, gender, KS, comorbidities, IFN-γ, IL-10, IL-2, IL-6, TNF-α, CD3, CD4, CD8, CD56, SARS-CoV-2-NAbs, CoV-2-STs, CPK, CRP, D-Dimers, ferritin, LDH, and WBC). Day 5 was prespecified as the early posttreatment anchor—the earliest uniform assessment across arms following therapy administration—because it captures the initial on-treatment biomarker dynamics and is the time point used for all prognostic models. Using baseline (day 0) and this posttreatment day-5 time point, we first computed the vector of 5-day changes (Δ=day 5–day 0) across biomarkers.

For the arm-swap Monte Carlo scenario analysis, day-5 biomarker values were not taken directly from the observed data but were generated from 4 variance models for the 5-day change rates: (1) additive independent and identically distributed (IID) bootstrap, (2) additive IID normal (clipped to the donor 1%‐99% range), (3) additive multivariate normal with a Ledoit-Wolf shrinkage covariance, and (4) multiplicative IID bootstrap on percent-per-day changes. Specifically, for each arm, we simulated day 5 values as (day 5_sim=day 0+Δ̃) where Δ̃ was drawn from the corresponding variance model for that arm. Simulated day-5 values were clipped to physiologic ranges when necessary. For each individual, we generated n=1000 Monte Carlo scenarios per arm. Each simulated day-5 profile was then scored with the donor arm’s LDA model, yielding a predicted probability of the critical class at the day-60 outcome, and for each patient, we stored the mean predicted probability across Monte Carlo simulations.

Swap summaries are evaluated at a single fixed threshold of 0.50. A 0.5 probability threshold is the standard “neutral” cutoff for binary classifiers and corresponds to a decision threshold where false positives and false negatives are weighted equally in simple cost terms. Using a constant 0.5 avoids threshold-tuning bias, sidesteps calibration differences between donor and recipient arms, and keeps the reported critical class easy to interpret. We provide baseline-risk quartile subgroup counts, and repeat the swap with CV-trained donor models to obtain mean and SD class-1 counts across the Monte Carlo simulations. Swapped-arm predictions are predictive scenario analyses, not causal effects.

**Figure 5.**
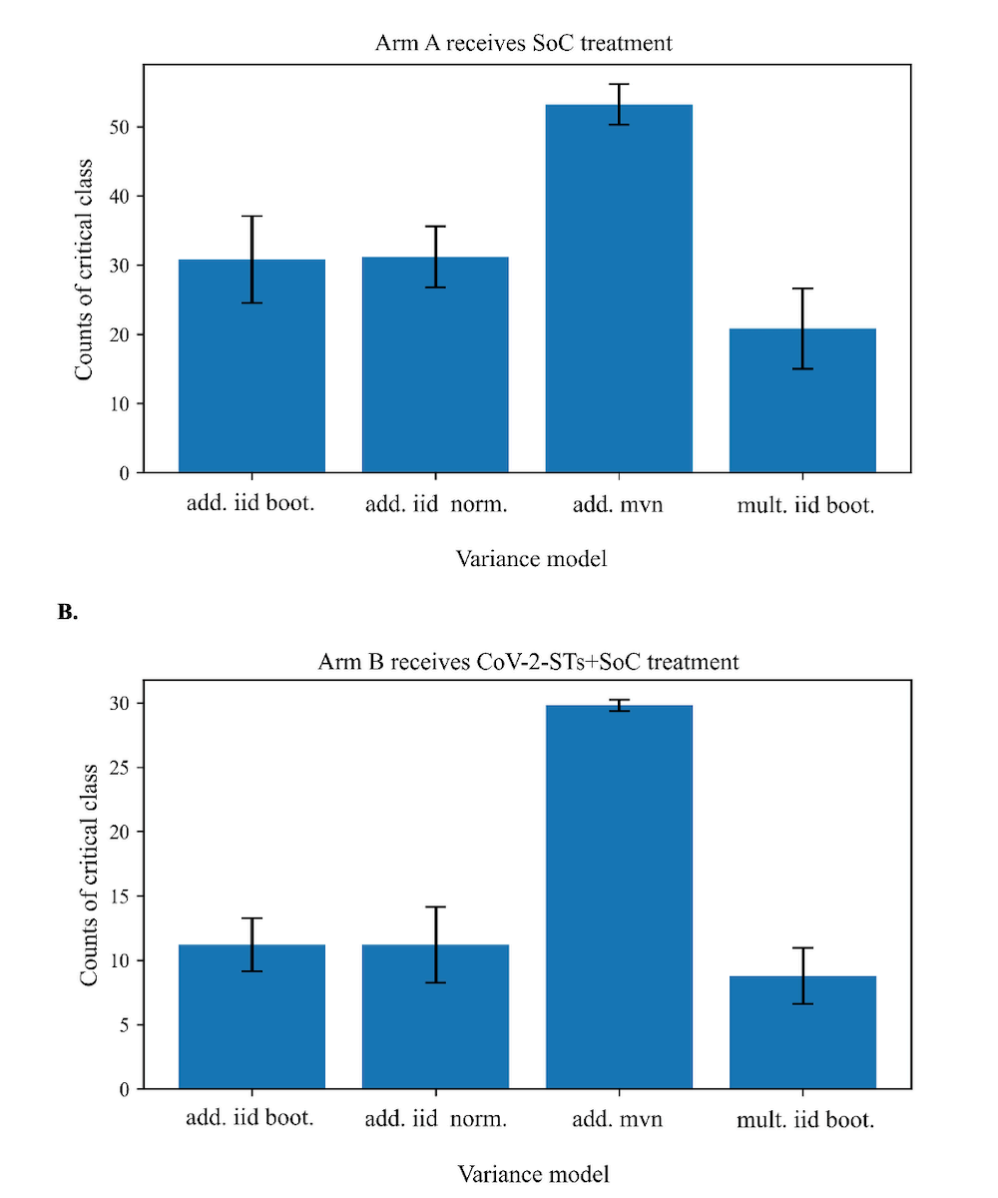
(A) Arm A (standard-of-care). Mean predicted count of critical-class patients at a fixed, neutral decision threshold 0.50. Bars correspond to 4 variance models for 0→5-day change-rates—additive iid bootstrap, additive iid normal (clipped 1%‐99%), additive mvn, and multiplicative iid bootstrap on percent-per-day. Predictions come from LDA shrinkage scored on Monte Carlo–simulated day-5 biomarkers; error bars represent SDs. (B) Arm B (SARS-CoV-2–specific T cell+standard-of-care). Same analysis as in panel A, that is, mean predicted critical-class counts at threshold 0.50 across the 4 variance models, with ±SD. add. iid boot.: additive independent and identically distributed bootstrap; add. iid norm: additive independent and identically distributed normal; add. mnv: additive multivariate normal; CoV-2-ST: SARS-CoV-2–specific T cell; mult. iid boot.: multiplicative independent and identically distributed bootstrap; SoC: standard-of-care.

When arm A receives SoC-like early dynamics (Figure 5A), at a stringent 0.5 cutoff, the predicted number in the critical class depends on the variance model but is always ≥ the observed 35.1%. With the multivariate normal (MVN) model, the model calls 54 (SD 3; across CV refits) out of 57 patients, corresponding to 94.7% of the cohort and approximately 34 additional patients relative to the observed outcome. With IID additive generators the increase is moderate, 31/57 (SD 6; 54.4% of the cohort; approximately 11 additional critical patients), and additive IID normal, 31 (SD 5) critical cases out of 57 patients (54.4% of the cohort; approximately 11 patients extra). With multiplicative IID, the estimate is 21 (SD 6) out of 57 (36.8% of the cohort; approximately 1 patient extra). SDs across 5-fold CV refits are small relative to the between-model gaps, indicating that modeling assumptions, not refitting noise, drive the shift at this threshold.

When arm B receives CoV-2-STs+SoC early dynamics, at a stringent 0.5 cutoff, the results are highly sensitive to correlation (Figure 5B). Under MVN, the model calls 30 (SD 1; across CV refits) out of 30 patients, corresponding to 100% of the cohort, and approximately 12 patients extra. Under IID additive assumptions, the predictions drop well below trial, 11 (SD 2) out of 30 patients (36.7% of the cohort; 7 patients less), and additive IID normal, 11 (SD 3) out of 30 patients (36.7% of the cohort; 7 patients less). With multiplicative IID, the estimate is 9 (SD 2) out of 30 patients (30% of the cohort; 9 patients less). Again, the SDs are small compared with the MVN-IID separation, underscoring that retaining cross-marker comovement is the key determinant at high thresholds. The divergence of the MVN model in arm B (predicting 100% critical outcomes) is likely attributable to the instability of estimating a high-dimensional covariance matrix from the small sample size (n=30), which can amplify noise. Consequently, the independent (IID) bootstrap models likely provide a more reliable estimate for this specific small-cohort scenario.

These are predictive scenario analyses, not causal effects. They assume harmonized day-0 measurements, transportability of the donor from day 0 to day 5, change distributions to recipients, and no interference between patients; thresholds are learned on the donor arm, so cross-arm counts should not be read as treatment effects. The sensitivity panels across the 4 variance models are therefore the right way to present robustness (from CoV-2-STs+Soc to SoC-only) and dependency (from SoC-only to CoV-2-STs+SoC) without making causal claims.

We need to acknowledge here that cellular immune responses are not routinely assessed in health care facilities. Therefore, we developed an alternative version of the tool to facilitate its broader applicability, which excludes the circulating CoV-2-STs. This model still maintains the same characteristics as the model including all biomarkers (Figure S4 and S5 in Multimedia Appendix 1).

## Discussion

### Principal Results

In previous studies, both our group and others have demonstrated a strong association between CoV-2-ST responses and favorable outcomes [12,38,39]. Furthermore, in a recent study, we showed that incorporating CoV-2-STs generated from recovered donors into the standard care regimen—including remdesivir and dexamethasone—markedly improved recovery and survival rates in patients with severe COVID-19, caused by the delta variant, compared with the SoC alone. This method of adoptive immunotherapy led to a rapid increase of circulating CoV-2-STs in vivo and contributed to the overall rebuilding of the immune system. Expanding on these findings, we here used ML for a post hoc analysis of the already-published clinical trial data to identify pivotal time points, characteristics, and biomarkers that are decisive for the outcome in severe COVID-19 cases under both treatment approaches [12]. Our analysis pinpointed day 5 post enrollment as a critical juncture at which distinct immune responses to CoV-2-ST administration compared with SoC become apparent, especially in the kinetics of T lymphocytes, circulating CoV-2-STs, cytotoxic T lymphocytes, and NK cells. This early distinction indicates a superior immune modulation by CoV-2-STs, impacting both innate and adaptive immunity, and corresponds with our clinical findings of faster recovery from lymphopenia, maintenance of innate immunity homeostasis, and enhancement of SARS-CoV-2–specific immunity in patients treated with CoV-2-STs in addition to SoC [12].

Furthermore, by using regularized or shrinkage LDA, we determined key predictors of patient outcomes by day 60, underlining the role of SARS-CoV-2–specific immunity in managing severe COVID-19 cases. Our LDA models, tailored for forecasting responses to CoV-2-STs+SoC or SoC-alone treatment, facilitated the development of a computational tool aimed at guiding therapeutic decisions. This tool, by considering initial patient characteristics and biomarker levels, seeks to identify individuals among those with severe COVID-19 who may not respond to SoC favorably, thus becoming prime candidates for alternative interventions, such as adoptive immunotherapy with CoV-2-STs. By simulating over 1000 potential disease progression profiles and using our models to compute survival probabilities, we present a new method for choosing treatment strategies that optimize patient outcomes. Testing this tool in hypothetical scenarios, we have further validated its potential for practical application, showcasing its adaptability to treatment modifications and its predictive accuracy across diverse patient responses, thereby highlighting its value in personalized medicine for severe COVID-19.

Our study benefits from the inclusion of a consistent sample of delta variant, high-risk individuals, mirroring real-world scenarios. Additionally, the availability of longitudinal, clinical, and laboratory data prospectively collected throughout our previous phase I and II clinical trial adds strength to our analysis, despite its post hoc nature. Leveraging these data, our prognostic approach enabled us to make early and precise estimates of mortality upon admission. Although reported ML models have provided promising prognostic implications, they are still hampered by certain limitations, most commonly the lack of capability to predict the outcome at, or early after, admission. Our rapid risk assessment tool addresses this gap, empowering health care professionals to identify patients who are highly susceptible to experiencing unfavorable outcomes under routine treatment alone. This allows for immediate and individualized interventions, such as improved care protocols and monitoring practices, and the consideration of administering CoV-2-STs. Notably, our research pioneers the integration of circulating CoV-2-STs into an ML-based predictive tool, enhancing the precision and applicability of our prognostic model. This approach aligns with the learning health system principles successfully implemented in large-scale COVID-19 surveillance registries [40].

### Limitations

Our study also has certain limitations that need to be acknowledged. First, the patient sample size was limited, as the original clinical trial was not specifically tailored to accommodate an ML analysis, whereas the retrospective nature of our research precluded prospective evaluation in patient cohorts. Because this is a post hoc analysis of a trial not purpose-built for ML, our findings should be interpreted as predictive and exploratory rather than causal or deployment-ready. To strengthen generalizability, we outline external and prospective validation in independent cohorts with a locked model, prespecified thresholds, and ongoing calibration monitoring. Second, since the initial trial only included severe cases, our tool’s evaluation was confined to the severe COVID-19 context, limiting its broader applicability. Furthermore, vaccination status was not included as a variable in our computational tool, due to the low vaccination rates among study patients (10/57, 17.5% of the CoV-2-ST group and 8/30, 26.7% of the SoC group). Since significant protection was offered by vaccination against severe illness, the majority of the eligible patients with severe COVID-19 were unvaccinated. To prevent potential bias stemming from underrepresented vaccinated patients, we chose not to include vaccination status in the tool. Future work should explore the incorporation of interoperable data standards to enhance reproducibility, as exemplified in large European vaccine trials [41].

While directly useful, the ML models depend heavily on the quality and breadth of the data used. If the data are not representative of the broader population or if key variables are missing, the predictions may not be accurate or generalizable. Accordingly, performance may be sensitive to the data context and temporal drift, and transportability beyond severe COVID-19 should be verified with temporal or external validation. Additionally, treatment outcomes can be influenced by multifactorial and dynamic patient conditions not fully captured in the model. The ML COVID-19 studies mentioned in the Introduction section use a variety of data types (eg, imaging and clinical parameters) and are often designed to process and analyze complex datasets quickly, providing critical insights in fast-moving clinical scenarios like a pandemic [19,20,23,24,42]. Their use of advanced algorithms can detect patterns not readily apparent to humans, which is a significant advantage in diagnostic and prognostic contexts. While our treatment comparison model provides specific actionable data for clinical decisions between 2 treatments, the broader ML studies offer insights into disease characterization, risk stratification, and initial management strategies. Future work will include prospective, preregistered evaluation and harmonized data standards to improve reproducibility and clinical uptake. A comprehensive approach in clinical settings might involve integrating insights from both types of models—using ML-driven diagnostics and risk assessments to initially guide treatment choices and then applying the treatment comparison model to select the optimal therapy based on clinical outcome predictions. In essence, while the referenced ML works provide broad diagnostic and prognostic tools useful in managing COVID-19, our model comparing treatments between CoV-2-STs and SoC offers targeted, actionable insights specifically designed to optimize therapeutic outcomes. Both have distinct roles in clinical decision-making, demonstrating the diverse applications of ML in health care.

### Comparison With Previous Work

This study focuses on the application of ML methods to COVID-19 clinical management, introducing several innovations. Previous models have successfully predicted mortality, severity, or intensive care needs based on early clinical parameters, imaging data, and laboratory results [20,23,24,42]. However, these models often rely on static baseline data and do not incorporate dynamic immune monitoring, particularly T cell–specific parameters. Most ML COVID-19 models focus on population-level predictions rather than individualized therapeutic decision-making, and few address which patients will benefit from emerging advanced therapies. The COVID-19 ML application focuses on clinical interpretability and direct applicability, using LDA instead of opaque prediction tools. LDA generates transparent decision boundaries and provides clear insights into feature contributions, making it suitable for structured biomedical datasets. Its reliance on linear combinations of input features and ability to identify discriminative variables make it suitable for structured biomedical datasets. This aligns with calls from the clinical ML community to prioritize explainable models that foster trust and adoption among health care practitioners [33,43,44]. The work combines ML, immunological profiling, and outcome simulation to enhance precision medicine in severe COVID-19.

### Future Work

Future research possibilities will prioritize ensemble strategies to improve the robustness and clinical utility of our COVID-19 prognostic models. First, we will evaluate bagging with gradient-boosted base learners alongside principled preprocessing (eg, outlier detection and information-gain feature selection) to handle small, noisy clinical cohorts [45]. In parallel, we will develop hybrid ensembles (stacking or soft-voting) that combine linear, tree-based, and margin classifiers to capture complementary signals across biomarkers [46]. All candidates will be benchmarked under an identical stratified CV with AUROC or AUPRC, Brier, calibration (pre-post isotonic), decision curves, and thresholded confusion matrices, followed by temporal or external validation to test transportability and drift. Finally, we will pair ensembles with clinician-oriented explainability (global or local summaries and reliability tables) and outline a prospective evaluation to assess workflow impact.

### Conclusions

In conclusion, we provide a user-friendly computational tool that integrates clinical, biochemical, and immunological features, facilitating timely and accurate risk-stratification of patients with severe COVID-19. This will enable clinicians to make informed clinical decisions upon admission, predicting either recovery or clinical deterioration and promptly intervening when necessary. Independent, continuous validation of our computational tool in prospective settings and larger clinical trials will confirm its predictive accuracy, effectiveness, and usefulness in guiding therapeutic decisions.

## Supplementary material

10.2196/78471 Multimedia Appendix 1 Additional tables, figures, cross-validation analyses, Monte Carlo simulation results, and implementation details of the machine learning–based therapeutic decision-support tool for severe COVID-19.

## Abbreviations

AI: artificial intelligence
AUPRC: area under the precision-recall curve
AUROC: area under the receiver operating characteristic curve
CoV-2-NAb: SARS-CoV-2–specific neutralizing antibody
CoV-2-ST: SARS-CoV-2–specific T cell
CRP: C-reactive protein
CV: cross-validation
ELISpot: enzyme-linked immunospot
IFN-γ: interferon-gamma
IID: independent and identically distributed
IL: interleukin
KS: Karnofsky score
KW: Kruskal-Wallis
LDA: linear discriminant analysis
LDH: lactate dehydrogenase
ML: machine learning
MVN: multivariate normal
NK: natural killer
OOF: out-of-fold
SMOTE: Synthetic Minority Oversampling Technique
SoC: standard-of-care
svd: singular value decomposition
TNF-α: tumor necrosis factor-α
WBC: white blood cell
WHO: World Health Organization
