# Supplementary material for: Early Immunological Biomarkers for Personalized Treatment Selection in Severe COVID-19: Post Hoc Machine Learning Analysis of a Randomized Clinical Trial
Source: JMIR Med Inform. 2026 Jun 4;14:e78471. doi: 10.2196/78471 (PMC13235983; doi:10.2196/78471)
Supplement: Multimedia Appendix 1 [file medinform-v14-e78471-s001.docx]

**Supplementary Material**

**Cross-Validated Predictive Modeling of Severe COVID-19: Day-5 Biomarkers for Day-60 Risk Stratification and Treatment Selection**

Symeon Savvopoulos^1,2^, Anastasia Papadopoulou^3^, Georgios Karavalakis^3^, Ioanna Sakellari^3^, Grigorios Georgolopoulos^4^, Christos D. Argyropoulos^5, 6, 7^, Evangelia Yannaki^3,4^*, Haralampos Hatzikirou^1,8^*

1. Department of Mathematics, Khalifa University, Abu Dhabi, United Arab Emirates.
2. Department of Chemical Engineering and Technology, Cracow University of Technology, Warszawska 24, 31-155 Krakow, Poland.
3. Hematology Department-Hematopoietic Cell Transplantation Unit, Gene and Cell Therapy Center, “George Papanikolaou” Hospital, 57010 Thessaloniki, Greece
4. Department of Medicine, University of Washington, Seattle, WA 98195, USA
5. Department of Chemical Engineering, King Fahd University of Petroleum & Minerals, Dhahran, 31261, Saudi Arabia.
6. Interdisciplinary Research Center for Refining & Advanced Chemicals, King Fahd University of Petroleum & Minerals, Dhahran 31261, Saudi Arabia.
7. Center for Integrative Petroleum Research, King Fahd University of Petroleum and Minerals, Dhahran 31261, Saudi Arabia.
8. Technische Univesität Dresden, Center for Information Services and High Performance Computing, Nöthnitzer Straße 46, P.O. Box: 01062, Dresden, Germany

*These authors participated equally

Corresponding author: Haralampos Hatzikirou

**1. Supplementary tables**

**Table S1.** LDA-identified critical features 60-day patient outcome for each patient cohort.

| Arm | Outcome | Important features driving the patient outcome |
| --- | --- | --- |
| A |  |  |
|  | 60-day | **PC1: I)** CRP, Karnf., CD3, CD4, LDH, circulating CoV-2-STs, CD56, Ferritin, CD8, age, WBC, Con2Abs, DD, CPK, IL10, IL6, IFNg, **II)** IL2, gender, TNFa, corm.  **PC2: I)** gender, CRP, DD, CD8, LDH, TNFa, CD3, Karnf., IL10, Ferritin, Con2Abs, CD56, WBC, CPK, IFNg, circulating CoV-2-STs, **II)** CD4, IL6, corm., age, IL2 |
| B |  |  |
|  | 60-day | **PC1: I)** CRP, Karnf., CD3, circulating CoV-2-STs, IL2, age, IL6, CD4, WBC, CD8, LDH, DD, IFNg, **II**) IL10, gender, TNFa, CD56, Ferritin, corm., CPK, Con2Abs  **PC2: I**) age, circulating CoV-2-STs, CD56, CD4, gender, Con2Abs, CPK, Karnf., IL2, corm., Ferritin, CD3, TNFa, CD8, **II**) CRP, DD, IFNg, IL6, WBC, IL10, LDH |

Underlined elements were significantly different by Kruskal-Wallis test (*P*<.05) for continuous variables, and chi square (*P*<.05) for categorical variables among patients of the same cohort classified as i) alive and well, ii) alive with disease and iii) dead. Each principal component (PC) is separated in two groups. Group-I includes the features with higher eigenvalue in the eigenvector (≥0.1), and the set II includes the features with smaller eigenvalue in the same eigenvector (<0.1). Abbreviations: CoV-2NAbs – SARS CoV Antibodies, IFN-γ – Interferon gamma, IL-10 – Interleukin 10, IL-2 – Interleukin 2, IL-6 – Interleukin 6, TNF-a – Tumor Necrosis Factor, CoV-2-STs – SARS CoV Specific T cells, CPK – Creatinine Phosphokinase, CRP – C Reactive Protein, LDH – Lactate Dehydrogenase, WBC – White Blood Cells, IQR – Interquartile range

**Table S2:** CoV-2-STs+SoC arm—Performance out-of-fold with 95% confidence intervals. Shrinkage-LDA utilizing three imbalance strategies: empirical priors, balanced priors, and SMOTE-OHE. Reported metrics include AUPRC (primary), ROC AUC, Sensitivity, Specificity, Precision, F1, and Brier, accompanied by 95% bootstrap confidence intervals (2,000 replicates) calculated on out-of-fold predictions; within each replicate, the F1-optimal threshold was recalibrated. The table additionally enumerates the OOF threshold along with the counts of TN, FP, FN, and TP.


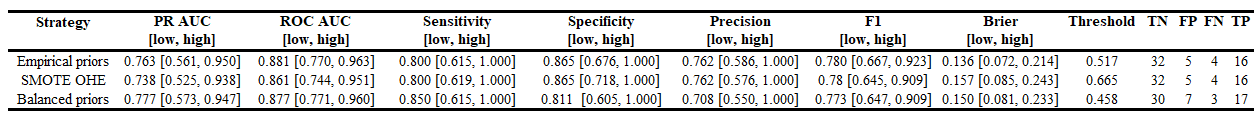


**Table S3:** CoV-2-STs+SoC arm—Consolidated validation confusion matrices for all cross-validation iterations.For each strategy, TN, FP, FN, and TP are aggregated across the validation folds (5×10 cross-validation). The derived rates (Sensitivity, Specificity, Precision, F1) from these aggregated counts are presented to enhance the OOF summaries.


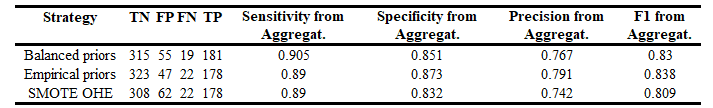


**Table S4:** CoV-2-STs+SoC arm—Per-fold validation performance (mean ± SD). For each strategy, fold-level AUPRC, ROC AUC, Brier, Accuracy, Precision, Sensitivity, Specificity, and F1 are summarized as mean ± SD across the 50 validation folds (5 folds × 10 repeats).


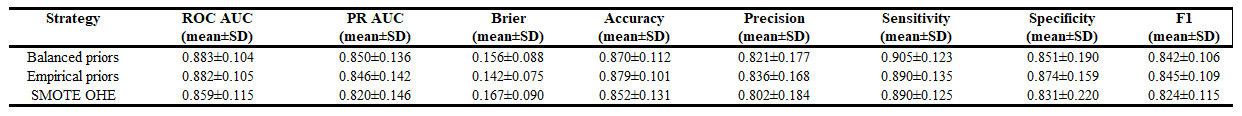


**Table S5:** SoC arm—Performance out-of-fold with 95% confidence intervals. Shrinkage-LDA utilizing three imbalance strategies: empirical priors, balanced priors, and SMOTE-OHE. Reported metrics include AUPRC (primary), ROC AUC, Sensitivity, Specificity, Precision, F1, and Brier, accompanied by 95% bootstrap confidence intervals (2,000 replicates) calculated on out-of-fold predictions; within each replicate, the F1-optimal threshold was recalibrated. The table additionally enumerates the OOF threshold along with the counts of TN, FP, FN, and TP.


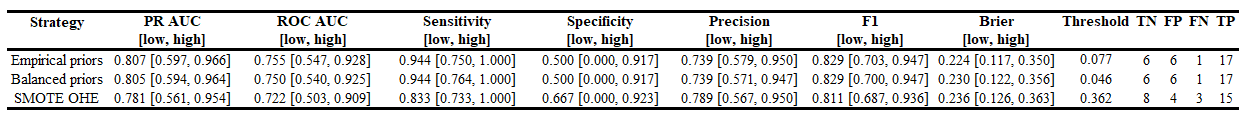


**Table S6:** SoC arm—Consolidated validation confusion matrices for all cross-validation iterations.For each strategy, TN, FP, FN, and TP are aggregated across the validation folds (5×10 cross-validation). The derived rates (Sensitivity, Specificity, Precision, F1) from these aggregated counts are presented to enhance the OOF summaries.


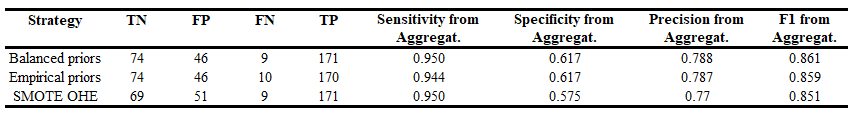


**Table S7:** SoC arm—Per-fold validation performance (mean ± SD). For each strategy, fold-level AUPRC, ROC AUC, Brier, Accuracy, Precision, Sensitivity, Specificity, and F1 are summarized as mean ± SD across the 50 validation folds (5 folds × 10 repeats).


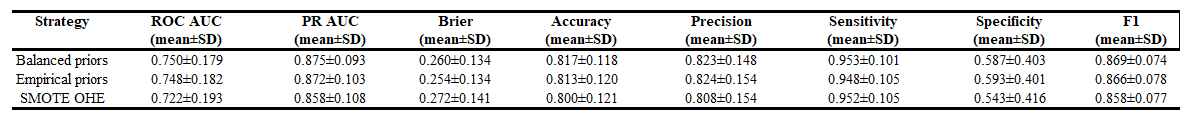


**2. Supplementary figures**

**
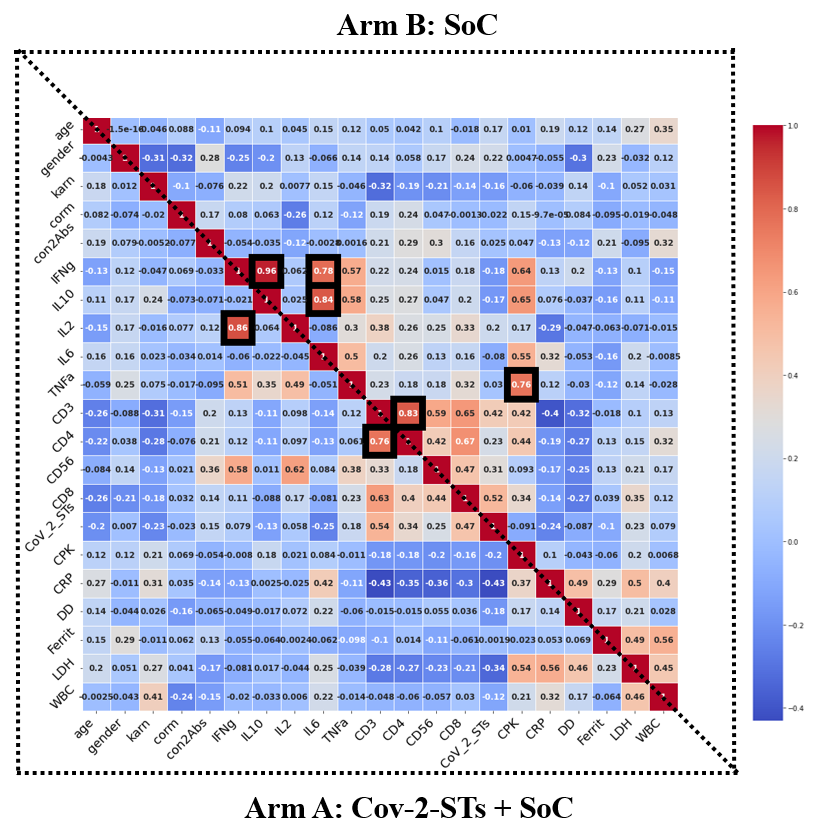
**

**Figure S1:** Correlation heatmap of CoV-2-STs+SoC and SoC groups at day 5.

| 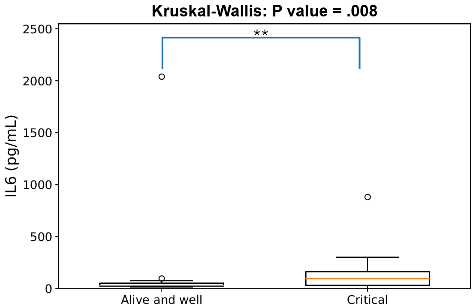 | 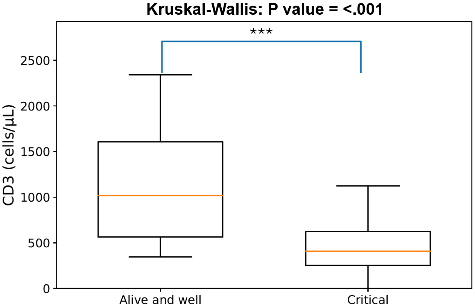 |
| --- | --- |
| 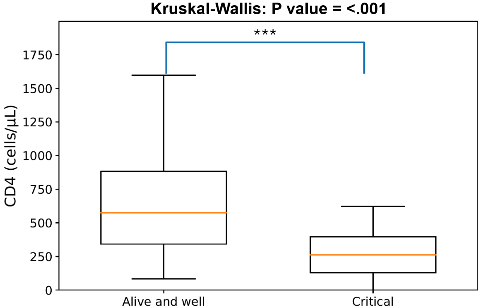 | 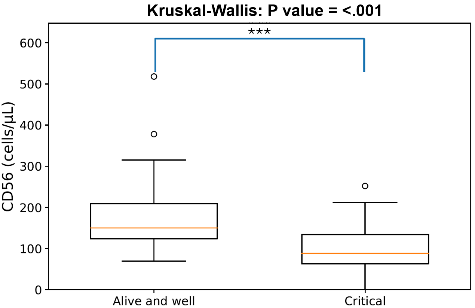 |
| 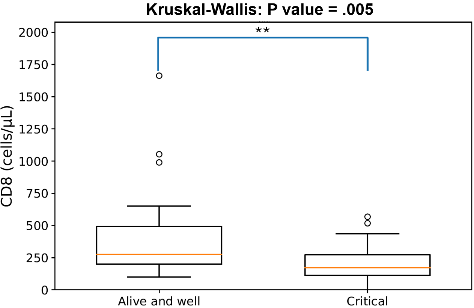 | 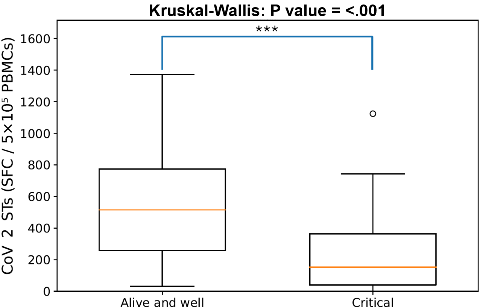 |
| 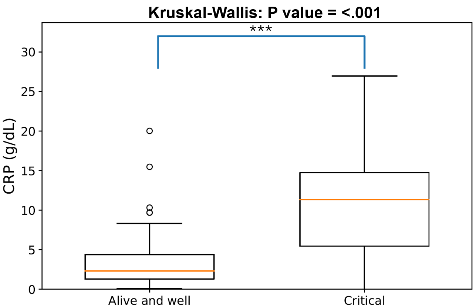 | 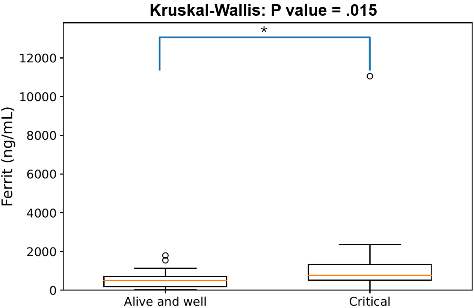 |
| 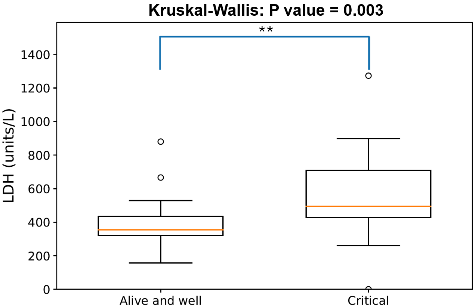 | 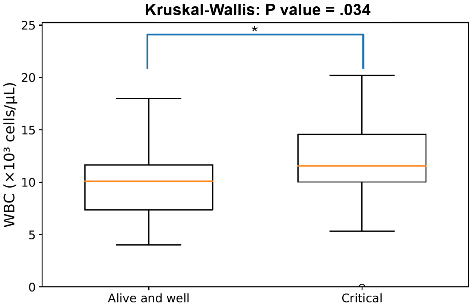 |
| 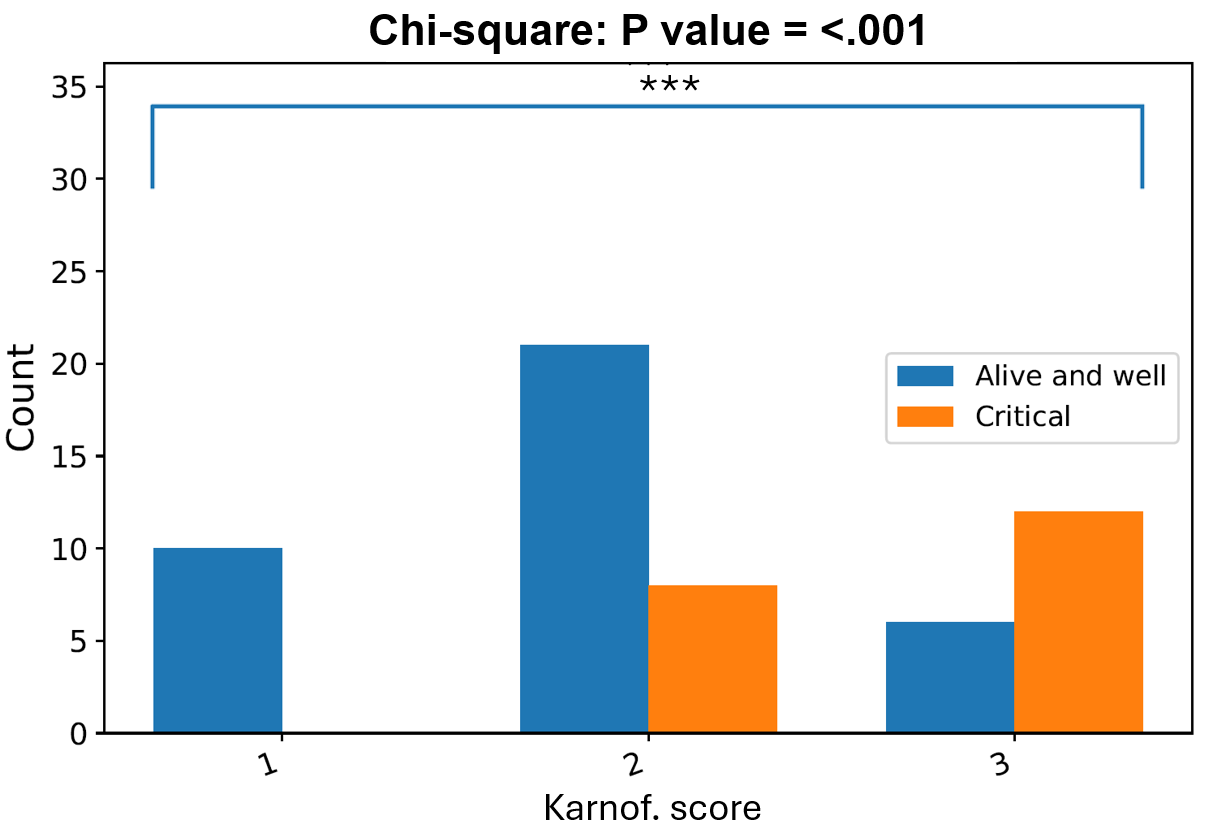 | |

**Figure S2:** The most critical features driving the 60-day clinical outcome of CoV-2-STs+SoC-treated patients, as identified by LDA. Differences between data sets were analyzed using Kruskal Wallis and Mann-Whitney with p<0.05, (*: P value<0.05, **: P value<0.01, ***: P value<0.001)

| 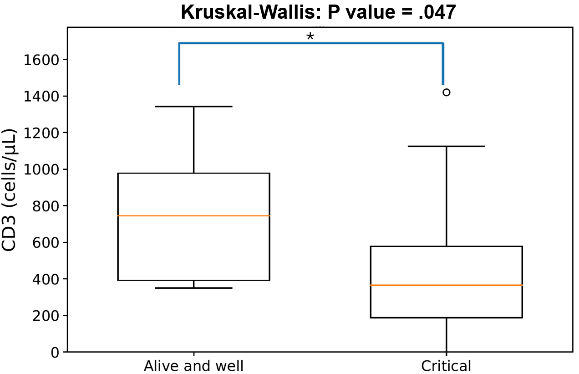 | 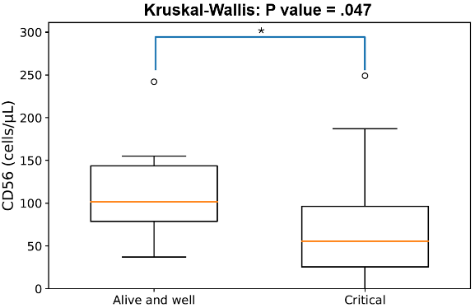 |
| --- | --- |
| 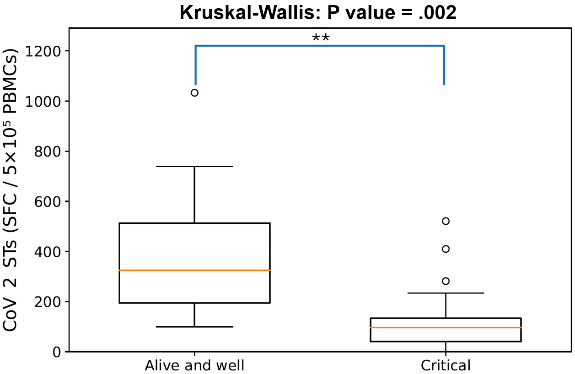 | 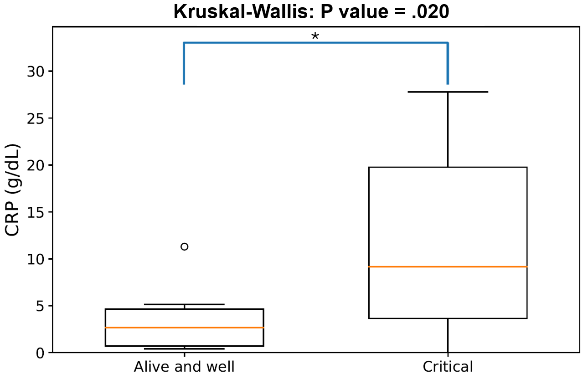 |

**Figure S3:** The most critical features driving the 60-day clinical outcome of SoC-treated patients, as identified by LDA. Differences between data sets were analyzed using Kruskal-Wallis and Mann-Whitney with p<0.05, (*: P value<0.05, **: P value<0.01, ***: P value<0.001)

| A. | B. |
| --- | --- |
| 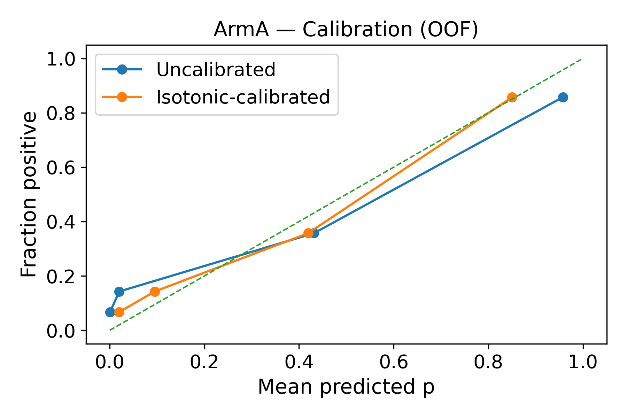 | 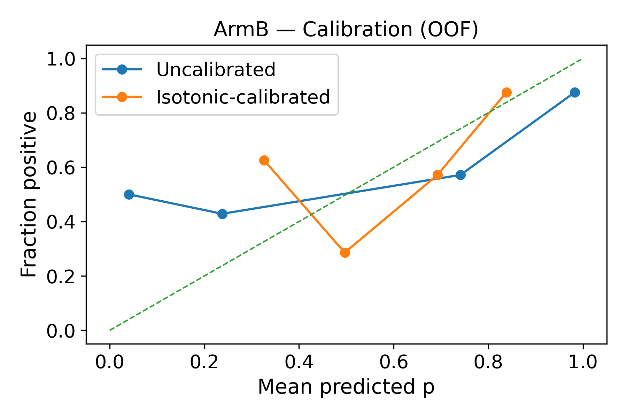 |
| C. | D. |
| 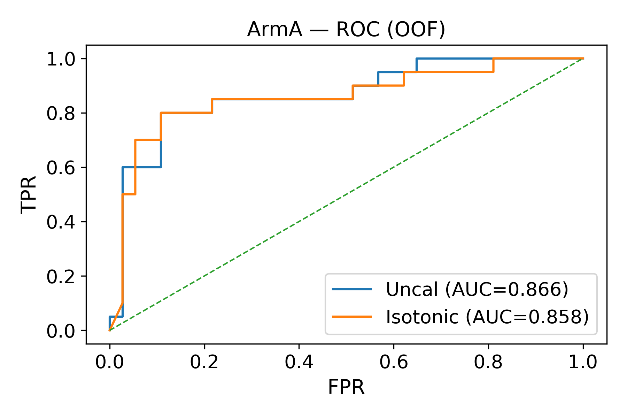 | 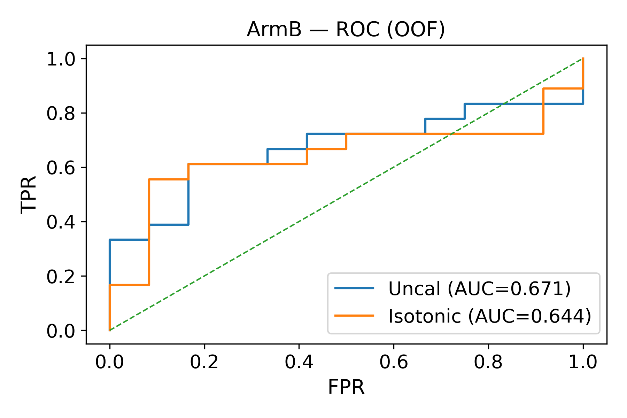 |
| E. | F. |
| 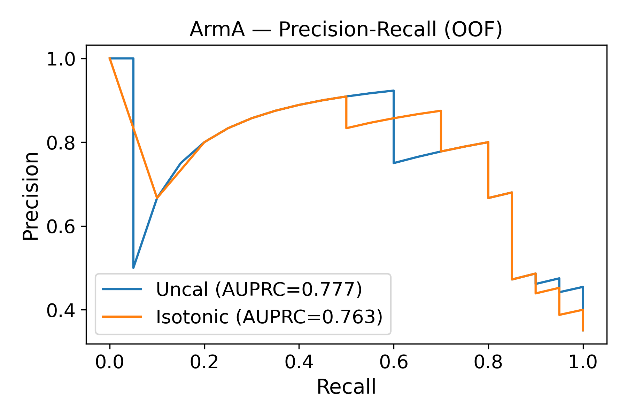 | 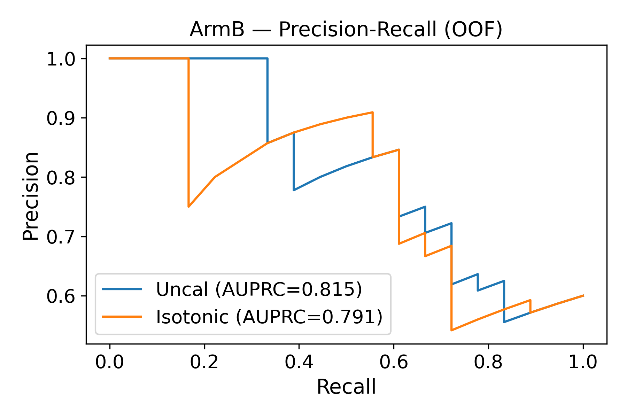 |
| G. | H. |
| 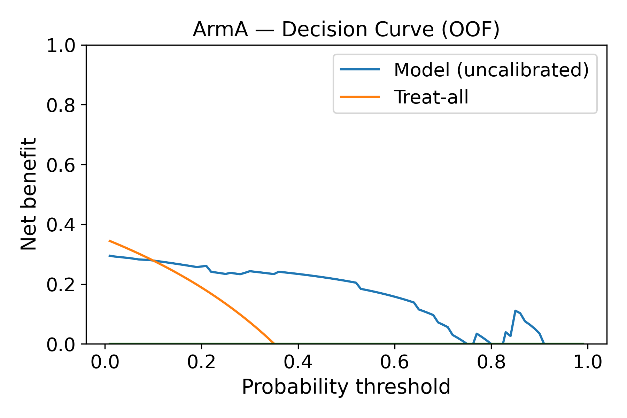 | 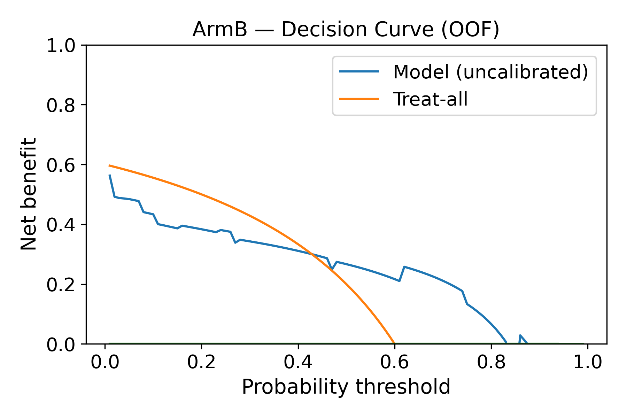 |

**Figure S4:** Out-of-fold performance visualizations. (A–B) Calibration (uncalibrated vs isotonic). (C–D) ROC curves with chance line. (E–F) Precision–recall curves. (G–H) Decision curves comparing the model with “treat-all” and “treat-none.” Left panels correspond to CoV-2-STs+SoC (Arm A); right panels to SoC (Arm B).

| **A.** |
| --- |
| 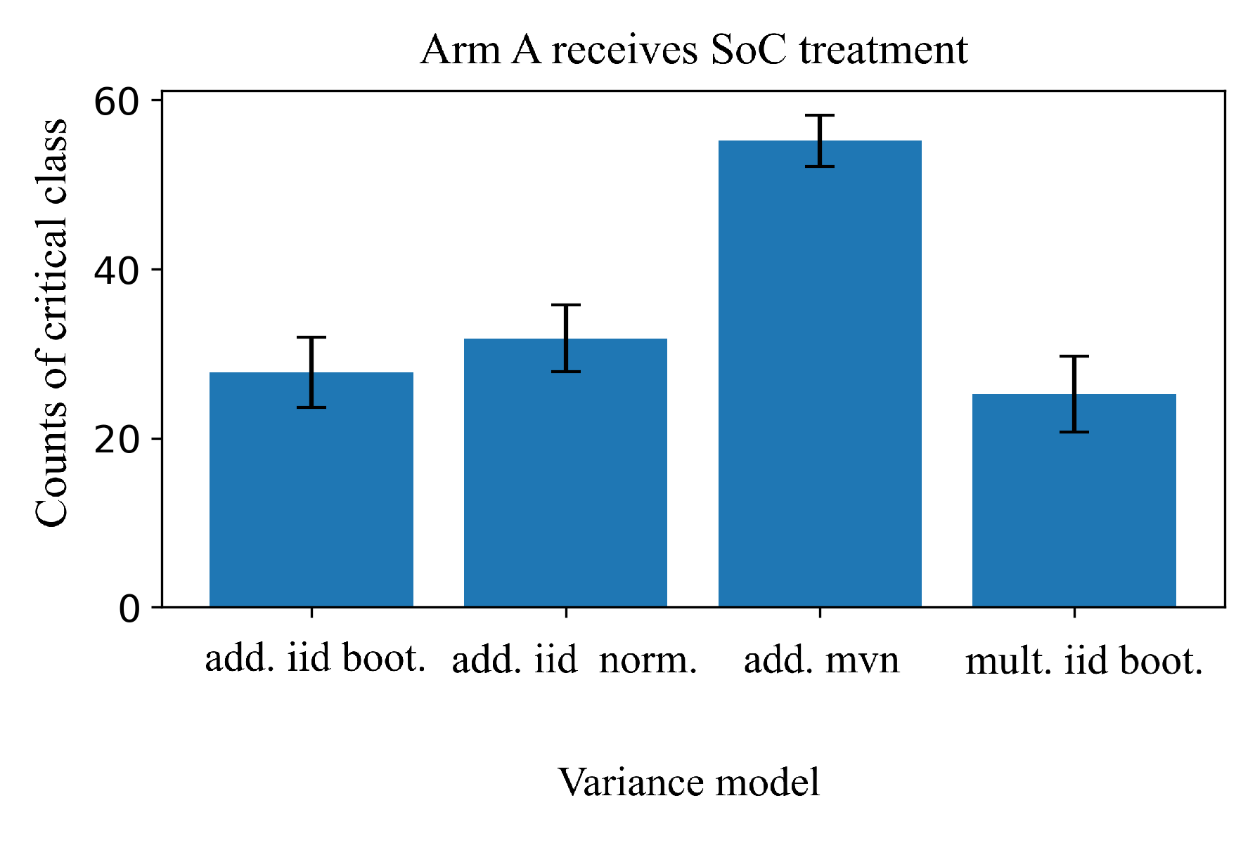 |
| **B.** |
| 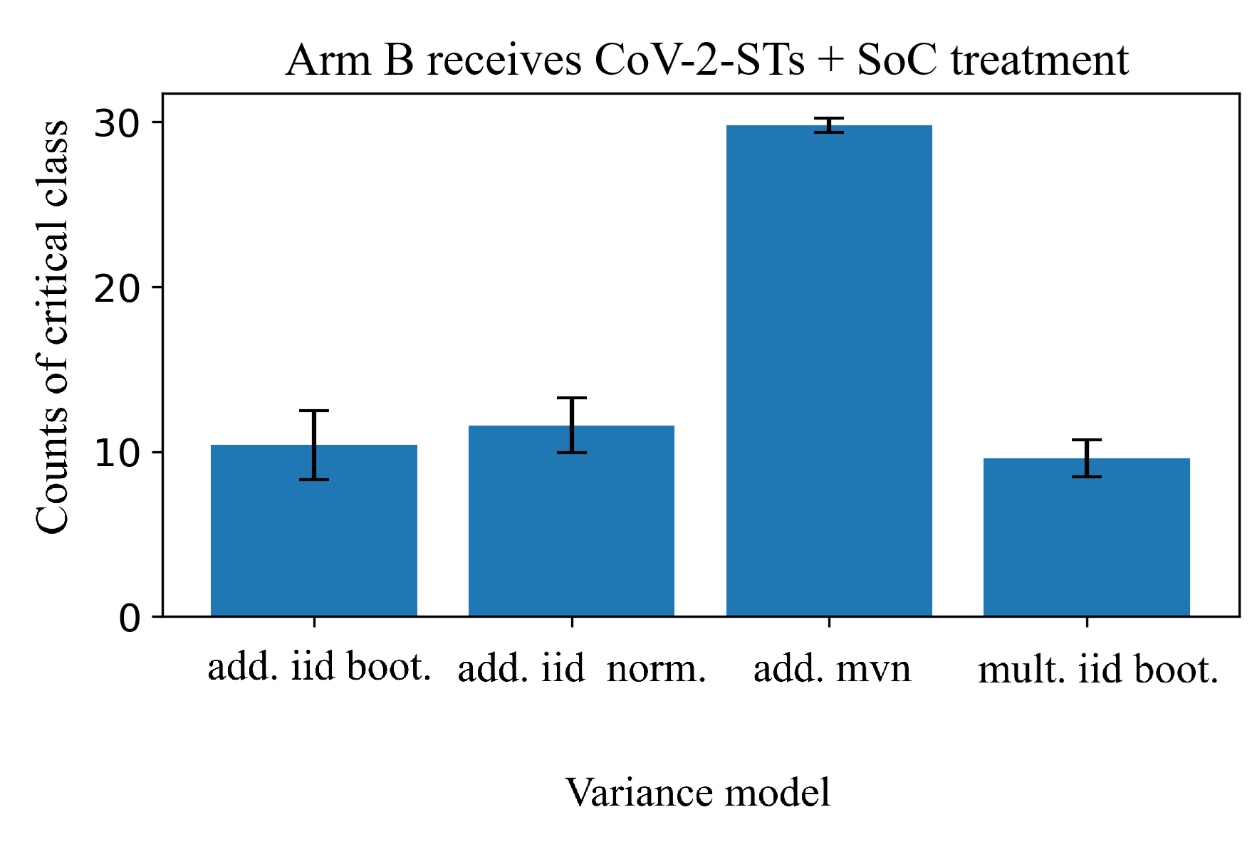 |

**Figure S5:** A) Arm A (SoC). Mean predicted count of critical-class patients at a fixed, neutral decision threshold 0.50. Bars correspond to four variance models for 0→5-day change-rates—additive iid bootstrap, additive iid Normal (clipped 1–99%), additive mvn, and multiplicative iid bootstrap on percent-per-day. Predictions come from LDA shrinkage scored on Monte-Carlo–simulated Day-5 biomarkers; error bars = ±SD. B) Arm B (CoV-2-STs + SoC). Same analysis as in Panel A: mean predicted critical-class counts at threshold 0.50 across the four variance models, with ±SD.

**3. Assessing treatment outcomes in hypothetical patient scenarios using our predictive decision-making tool**

A user-friendly app in Microsoft Excel designed for healthcare professionals managing COVID-19 patients. This innovative tool uses advanced LDA algorithms to predict patient outcomes under different treatment protocols, such as CoV-2-STs+SoC, or SoC only. This app offers personalized, data-driven insights to guide clinical decision-making. Developed with a comprehensive database of COVID-19 treatment outcomes and validated through clinical research, this app is not only user-friendly, but also compliant with healthcare privacy standards. This app aims to enhance treatment strategies, improve patient outcomes, and support healthcare providers in navigating the complexities of COVID-19 treatment.

In the first step (Figure S6), the only factors that need to be considered are individual patient characteristics at the time of diagnosis (day-0). These include age, gender, Karnofsky score, any comorbidities, and specific biomarkers and clinical parameters such as con2Abs, IFNg, IL10, IL2, IL6, TNFa, CD3, CD4, CD56, CD8, CoV-2-STs, CPK, CRP, D-Dimer DD, Ferritin, LDH, and WBC.


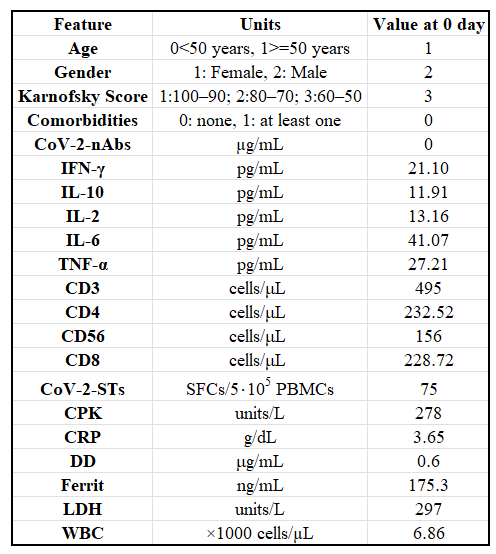


**Figure S6:** The entry of patient demographic and clinical characteristics at the baseline.

The user can activate the ML model after entering patient-specific measurements on day-0 (Figure S6 and S7). A personalized Monte Carlo scenario comparison is performed between two treatment arms, Arm A and Arm B, under four different variance models for 5-day biomarker evolution. The simulation is driven by a user-specified baseline profile and empirical 0–5 day biomarker change rates estimated from the trial data. A personalized baseline profile is first constructed from a CSV file (NEW_PATIENT_0d.csv). The 0 to 5-day change in biomarkers is characterized using aligned rate tables, which are derived from the corresponding Day-0 and Day-5 arm datasets. For each biomarker, these tables contain both absolute daily changes and proportional daily changes. Four distinct variance models are implemented to describe possible 5-day biomarker trajectories starting from the personalized baseline. In the non-parametric additive change model with independent and identically distributed bootstrap draws for daily absolute changes (add iid boot model), absolute daily change rates are sampled from donors via a non-parametric bootstrap and applied additively over 5 days. In the parametric additive change model with independent and identically distributed Gaussian draws for daily absolute changes (add iid norm model), rates are sampled from a normal distribution parameterized by the donor mean and standard deviation and truncated to the 1st–99th donor percentiles. In the correlated additive change model with multivariate normal draws for the vector of daily absolute changes across biomarkers (add mvn model), correlated additive rates are drawn from a multivariate normal distribution whose covariance matrix is estimated with Ledoit–Wolf shrinkage. In the non-parametric multiplicative change model with independent and identically distributed bootstrap draws for daily proportional (percent) changes (mult iid boot model), proportional daily changes are bootstrapped and applied multiplicatively to the baseline biomarkers across 5 days. For each variance model, the code performs 𝑅 Monte Carlo draws of complete 5-day biomarker profiles.

Each simulated 5-day biomarker profile is then scored with the appropriate arm-specific LDA shrinkage pipeline, yielding a predicted probability for the event class; the complementary alive and well class. For each Monte Carlo draw, the script counts how many patients are in alive and well class using a fixed decision threshold of 0.50 on the predicted event probability. This produces, for each combination of arm and variance model, a distribution of alive and well counts over the Monte Carlo replicates. The script summarizes these distributions in a table containing, for each arm and variance model, the mean number of alive and well patients, the corresponding standard deviation, and the total number of patients.

The script additionally performs Mann–Whitney U tests to compare the distributions of alive and well counts between arms for each variance model. For each model, a two-sided P-value and the two-sided P values are shown above the corresponding pair of bars.


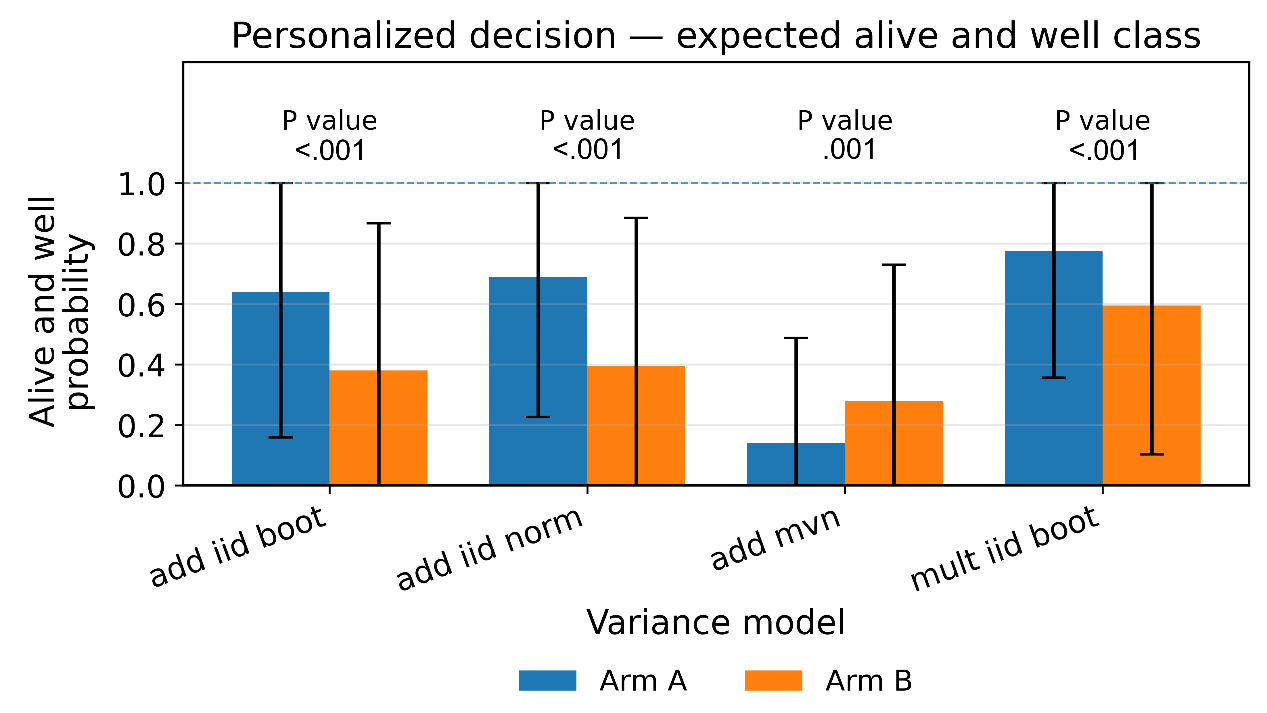


**Figure S7:** The personalized app conclusions under different treatment options.
